# Investigating Gold Deposition with High-Power Impulse Magnetron Sputtering and Direct-Current Magnetron Sputtering on Polystyrene, Poly-4-vinylpyridine, and Polystyrene Sulfonic Acid

**DOI:** 10.1021/acs.langmuir.4c02344

**Published:** 2024-10-15

**Authors:** Yusuf Bulut, Benedikt Sochor, Kristian A. Reck, Bernhard Schummer, Alexander Meinhardt, Jonas Drewes, Suzhe Liang, Tianfu Guan, Arno Jeromin, Andreas Stierle, Thomas F. Keller, Thomas Strunskus, Franz Faupel, Peter Müller-Buschbaum, Stephan V. Roth

**Affiliations:** † Deutsches Elektronen-Synchrotron DESY, Notkestr. 85, Hamburg 22607, Germany; ‡ Department of Physics, Chair for Functional Materials, Technical University of Munich, TUM School of Natural Sciences, James-Franck-Str. 1, Garching 85748, Germany; § Chair for Multicomponent Materials, Department for Materials Science, Faculty of Engineering, Kiel University, Kaiserstr. 2, Kiel 24143, Germany; ∥ Fraunhofer Institute for Integrated Circuits IIS, Development Center for X-ray Technology EZRT, Flugplatzstr. 75, Fürth 90768, Germany; ⊥ Centre for X-ray and Nano Science CXNS, Deutsches Elektronen-Synchrotron DESY, Notkestr. 85, Hamburg 22607, Germany; # Department of Physics, University of Hamburg, Notkestr. 9-11, Hamburg 22607, Germany; ∇ Heinz Maier-Leibnitz Zentrum (MLZ), Technical University of Munich, Lichtenbergstraße 1, Garching 85748, Germany; ○ KTH Royal Institute of Technology, Teknikringen 56-58, Stockholm 100 44, Sweden

## Abstract

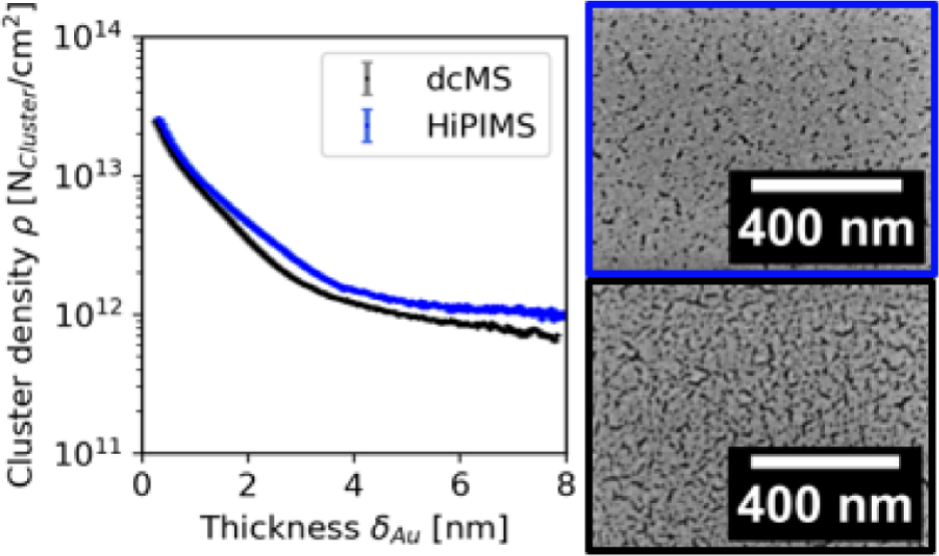

Fabricating thin metal layers and particularly observing their formation process in situ is of fundamental interest to tailor the quality of such a layer on polymers for organic electronics. In particular, the process of high power impulse magnetron sputtering (HiPIMS) for establishing thin metal layers has sparsely been explored in situ. Hence, in this study, we investigate the growth of thin gold (Au) layers with HiPIMS and compare their growth with thin Au layers prepared by conventional direct current magnetron sputtering (dcMS). Au was chosen because it is an inert noble metal and has a high scattering length density. This allows us to track the growing nanostructures via grazing incidence scattering. In particular, Au deposition on the polymer polystyrene (PS) with the respective structural analogues poly-4-vinlypyridine (P4VP) and polystyrene sulfonic acid (PSS) is studied. Additionally, the nanostructured layers on these different polymer films are further probed by field emission scanning electron microscopy (FESEM), atomic force microscopy (AFM), X-ray reflectometry (XRR), and four-point probe measurements. We report that HiPIMS leads to smaller island-to-island distances throughout the whole sputter process. Moreover, an increased cluster density and an earlier percolation threshold are achieved compared to dcMS. Additionally, in the early stage, we observe a significant increase in coverage by HiPIMS, which is favorable for the improvement of the polymer–metal interface.

## Introduction

Functional materials are of vital interest for various applications including sensors, batteries, and wearables.^1−3^ The class of polymer-based applications increased in recent decades; especially, the usage of polymer–metal hybrid materials gained attention.^4−7^ Gold is favorable and is used as a material for photoluminescence,^8^ plasmonics,^9−12^ mirrors,^13−15^ catalysis,^16−19^ sensors,^20−22^ solar cells,^23−26^ and electrodes.^27−30^ Direct current magnetron sputtering (dcMS) is a well-established and well-understood technique that is being used for fabricating thin metal layers in the aforementioned applications. However, the favorable physical properties of the metal layer are still not obtained in a single step since a preprocessing step like a plasma pretreatment is required to increase the adhesion in dcMS.^31,32^ This is undesirable if metal–polymer hybrid structures are fabricated using substrates being sensitive toward these preprocessing treatments. One emerging physical vapor deposition technique to overcome these pretreatments is high-power impulse magnetron sputtering (HiPIMS).^31,32^ This technique increases the adhesion of metal–polymer composites while being benign for heat sensitive substrates. Grazing incidence small angle X-ray scattering (GISAXS) and grazing incidence wide angle X-ray scattering (GIWAXS) studies were conducted to understand the formation behavior of dcMS-deposited metals films, but sparse studies were undertaken to understand the in situ film formation of HiPIMS deposited metals.^28,33^ Under dcMS and HiPIMS conditions, ionized and nonionized metal species are formed simultaneously during ablation at the sputter target. Depending on the target material, dcMS achieves a degree of ionization of up to 10%, and in the case of HiPIMS, the fraction of the ionized species condensing on the substrate can make up between 50 and 90%.^31,34^ Thus, it is important to obtain similar sputter conditions of both dcMS and HiPIMS, e.g., sample-to-target distance sputter target, substrate, pressure, and sputter rate, in order to understand the differences in the underlying physical film formation.^35^ Particularly, changing the sputter rate has an influence on the percolation threshold, which was made sure to be similar in this study.^36−38^ This focuses on the quantification of the differences in depositing Au with dcMS and HiPIMS on three different polymers, namely, polystyrene (PS), poly-4-vinylpyridine (P4VP), and polystyrene sulfonic acid (PSS). These polymers differ in their structure and have different functional groups apparent. PS is a one-dimensional polymer chain consisting of a substituted polyethylene structure with pendant aromatic rings being phenyl groups as functional groups. PS is a well-known polymer and was previously used in former studies as a standard reference substrate to compare the growth of metals with other polymers.^38,39^ P4VP is like PS, a one-dimensional polymer chain consisting of a substituted polyethylene structure with pendant aromatic rings consisting of pyridine as a functional group. Furthermore, PSS is a one-dimensional polymer chain consisting of a substituted polyethylene structure with pendant aromatic rings consisting of a sulfonic acid functional group on the phenyl group located on the *para* position. Due to different functional groups being apparent in the polymeric structure, it is expected that the different chemical structures change the surface energy of the polymers and thus particularly influence the growth of gold during deposition. The skeletal structure of the polymer can be found in Figure S1. The morphology of Au deposition via dcMS and HiPIMS on PS, P4VP, and PSS is investigated by field emission scanning electron microscopy (FESEM), atomic force microscopy (AFM), grazing incidence small-angle X-ray scattering (GISAXS), grazing incidence wide-angle X-ray scattering (GIWAXS), X-ray reflectometry (XRR), and four-point-probe measurements. We observe in situ the structural evolution of the Au cluster during dcMS and HiPIMS deposition on the polymers PS, P4VP, and PSS. We show that an increase of Au coverage can be achieved with HiPIMS.

## Experimental Section

### Materials

Silicon wafers (Si-Mat Silicon Materials, Germany) were cut into 12 mm × 15 mm-sized pieces. They were cleaned in acidic bath containing a ratio of hydrogen peroxide (30%, Carl Roth) to sulfuric acid (96%, Carl Roth) of 1:2.2 at 70 °C for 15 min. After the cleaning procedure, the silicon wafer was rinsed with ultrapure water and stored in an ultrapure water bath. Polystyrene (PS, *M*_n_ = 62.0 kg/mol, PDI = 1.04), poly-4-vinylpyridine (P4VP, *M*_n_ = 77.5 kg/mol, PDI = 1.05), and polystyrene sulfonic acid (PSS, *M*_n_ = 62.0 kg/mol, PDI = 1.02) were supplied by Polymer Source (Canada). PS was dissolved in toluene with a concentration of *c*_PS_ = 9 mg/mL, poly-4-vinylpyridine was dissolved in dimethylformamide with a concentration of *c*_P4VP_ = 26 mg/mL, and polystyrene sulfonic acid was dissolved in ultrapure water with a concentration of *c*_PSS_ = 25 mg/mL. PS and P4VP solutions were heat treated for 2 h at 70 °C, and PSS solution was heat treated at 90 °C for 15 min and stirred afterward for 2 h at RT.

### Polymer Film Preparation

The precleaned silicon wafer pieces were removed from the storage bath and rinsed with ultrapure water, which was followed by blowing them dry with a nitrogen steam. These silicon pieces were loaded into a spin coater (6-RC, SÜSS MicroTec Lithography, Germany). For PS and P4VP, 3600 rpm was used with the ramp-up setting of 9 for 30 s. For PSS, 6000 rpm was chosen with a ramp up setting of 9 for 30 s to obtain smooth thins films. The ramp-up setting of 9 corresponds to the highest acceleration rate allowed by the spin coater reaching the targeted rotation speed. The spin-coated polymer films were further used as spun in the following procedures. The thickness of the polymer films was determined by X-ray reflectometry (XRR) (Figure S2) with the corresponding SLD profiles shown in Figure S3 as δ_PS_ = 39 ± 1 nm for PS, δ_P4VP_ = 40 ± 1 nm for P4VP, and δ_PSS_ = 28 ± 1 nm for PSS. The fits are displayed in Figure S2 and the SLD curves are displayed in Figure S3. The film thicknesses are large enough to neglect potential influences of the substrate (Si/SiO_2_).^40^

### Physical Vapor Deposition

The self-built sputter deposition chamber was described in a previous article.^38^ For all experiments, a 2 in. size Au (Kurt J. Lesker, purity 99.999%) target was used. The dynamic working pressure was *p*_Ar_ = 0.36 Pa with an argon flow adjusted to 10 sccm. The average power for dcMS was *P*_dcMS_ = 27 W. For HiPIMS, deposition was performed at *P*_HiPIMS_ = 40 W with the pulse length being 50 μs and the frequency being 1 kHz. The chosen dcMS and HiPIMS conditions were stable throughout the experiment. In case of dcMS, the average kinetic energy of the sputtered atoms is 3.9 ± 0.1 eV. The average kinetic energy of the sputtered atoms for HiPIMS is 9.7 ± 0.1 eV having a non-Gaussian distribution. The average kinetic energy of the sputtered atoms for both sputtering modes were measured with a Quantum Probe (Impedans, Ireland). The distance between the target and sample was 13 cm. The deposition rates were determined by a quartz crystal microbalance (QCM) being 0.294 ± 0.006 nm/s for HiPIMS and dcMS. The QCM deposition rates were verified by measuring final thicknesses of deposited gold films on silicon with a profilometer (Dektak XT, Bruker). In addition, the last detector frames of the in situ sequence were used by extracting the distance of adjacent vertical peaks in the *q*_*z*_ direction with the relation δ = 2π/Δ*q*_*z*_ to determine the final deposited thickness for all in situ thicknesses.^41^ The last detector frames of the in situ sequences are displayed in Figure S4. Additionally, XRR was performed before and after the in situ deposition on the polymers PS, P4VP, and PSS, which are shown in Figure S2 for the pristine polymers and in Figure S5 for the after in situ deposition.

### Characterization

#### Field Emission Scanning Electron Microscopy

High-resolution field emission scanning electron microscopy (FESEM) images were taken with a Nova Nano SEM 450 (FEI Thermofisher) at DESY NanoLab.^42^ For the visualization of the images, the software ImageJ was used.^43^ The nanoparticle size was determined with ImageJ by counting only individual clusters that were not connected and measured manually. FESEM images of the pristine thin films are displayed in Figure S6. No difference in surface morphology is observed for the pristine polymer films. In a 100 nm × 100 nm frame, the size of clusters is summarized in Table S1.

#### X-ray Scattering

The grazing-incidence small-angle X-ray scattering (GISAXS), grazing-incidence wide-angle X-ray scattering, and X-ray reflectometry experiments were conducted at DESY (P03/PETRA III, Hamburg, Germany) with a mobile sputter deposition chamber, which was previously reported.^38^ A wavelength of λ = 0.105 nm was chosen. For the measurements, the X-ray beam had a point shape with a size of 30 μm × 25 μm with an incident angle α_I_ = 0.4°. For GISAXS, a Pilatus 2 M (pixel size 172 μm, Dectris Ltd., Switzerland) was placed at a distance to the sample of SDD_GISAXS_ of 3230 ± 2 mm. The data acquisition rate was set for HiPIMS to 20 Hz and that for dcMS to 10 Hz. Raw GISAXS images are displayed in the Supporting Information for Au:PS_dcMS_ (Figure S7), Au:PS_HiPIMS_ (Figure S8), Au:P4VP_dcMS_ (Figure S9), Au:P4VP_HiPIMS_ (Figure S10), Au:PSS_dcMS_ (Figure S11), and Au:PSS_HiPIMS_ (Figure S12). For GIWAXS, a Lambda 9 M (X-Spectrum, Germany) was used with the distance to the sample being SDD_GIWAXS_ = 193 ± 2 mm. For HiPIMS deposition, the data acquisition rate was set to 2 Hz, and for dcMS, it was set to 1 Hz. The pixel size of one pixel of the Lambda 9 M is 55 μm × 55 μm. Raw GIWAXS images are displayed in the Supporting Information for Au:PS_dcMS_ (Figure S13), Au:PS_HiPIMS_ (Figure S14), Au:P4VP_dcMS_ (Figure S15), Au:P4VP_HiPIMS_ (Figure S16), Au:PSS_dcMS_ (Figure S17), and Au:PSS_HiPIMS_ (Figure S18). The software DPDAK was used for analyzing the measured GISAXS and GIWAXS data.^44^ X-ray reflectometry (XRR) measurements were performed with the aforementioned energy and were analyzed with MOTOFIT 0.1.20.^45^

#### Atomic Force Microscopy

The atomic force microscopy (AFM) measurements were taken with a Bruker (Dimension Icon equipped with a NanoScope V controller) at DESY NanoLab.^42^ RTESPA-150 (Bruker) cantilevers were used having a nominal tip radius of 8 nm. The software NanoScope Analysis was used for data visualization, and with ImageJ, the scale bar was included.

#### Four-Point Probe Measurements

Four-point probe measurements (Ossila, UK) were conducted with a probe spacing of 1.27 mm. The measurement range is between 100 mΩ/square and 10 MΩ/square. After 578 days, the samples were measured again and summarized in Table S2.

## Results and Discussion

**Figure 1:**
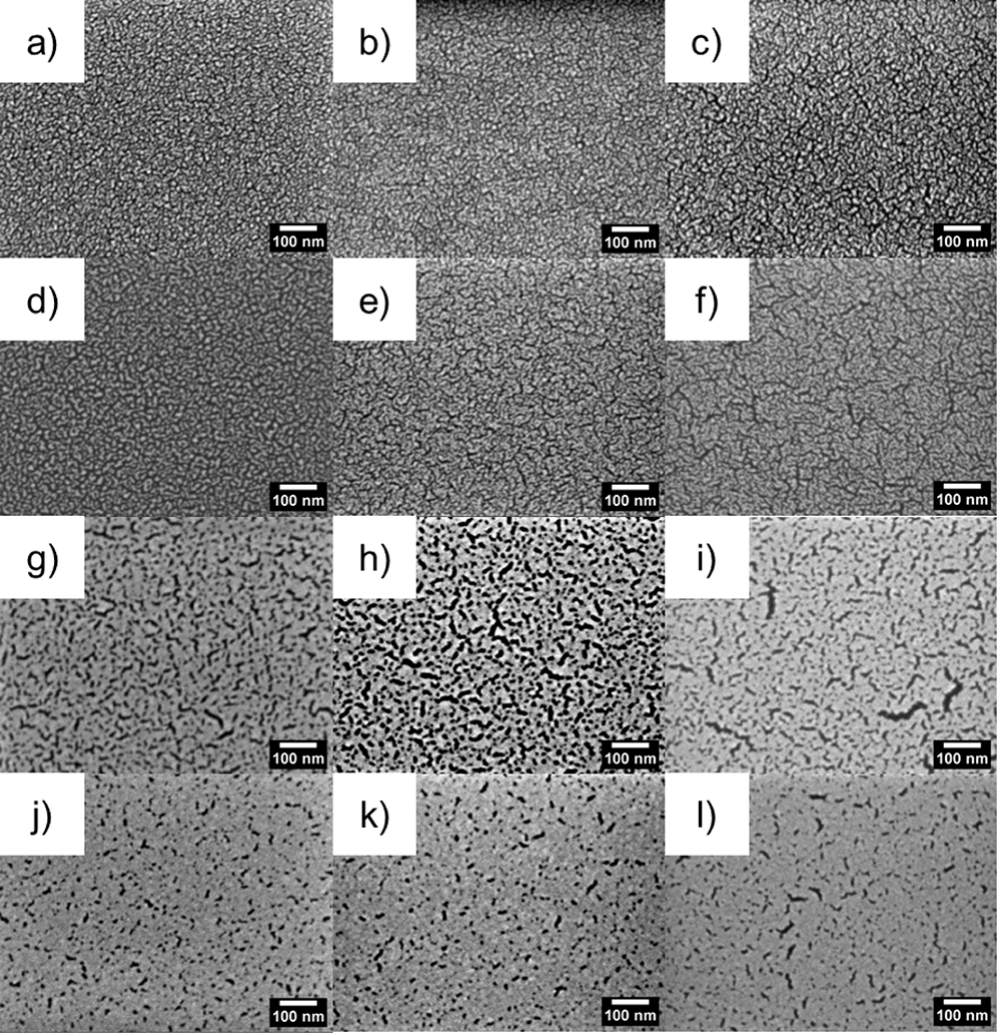
FESEM images of Au-coated (a, d, g, j) Au:PS, (b ,e, h, k) Au:P4VP, and (c, f, i, l) Au:PSS. Panels a–c were coated with δ_Au,dcMS_ = 2 nm, panels d–f were coated with δ_Au,HiPIMS_ = 2 nm, panels g–i were coated with δ_Au,dcMS_ = 4 nm, and panels j–l were coated with δ_Au,HiPIMS_ = 4 nm.

Au was deposited by direct current magnetron sputtering (dcMS) and by high power impulse magnetron sputtering (HiPIMS) on three different polymer films (PS, P4VP, and PSS). The morphologies of the Au layer at selected thicknesses (δ_Au_ = 2 and 4 nm) for both dcMS and HiPIMS are shown in the FESEM images in Figure 1 for PS, P4VP, and PSS.

**Figure 2:**
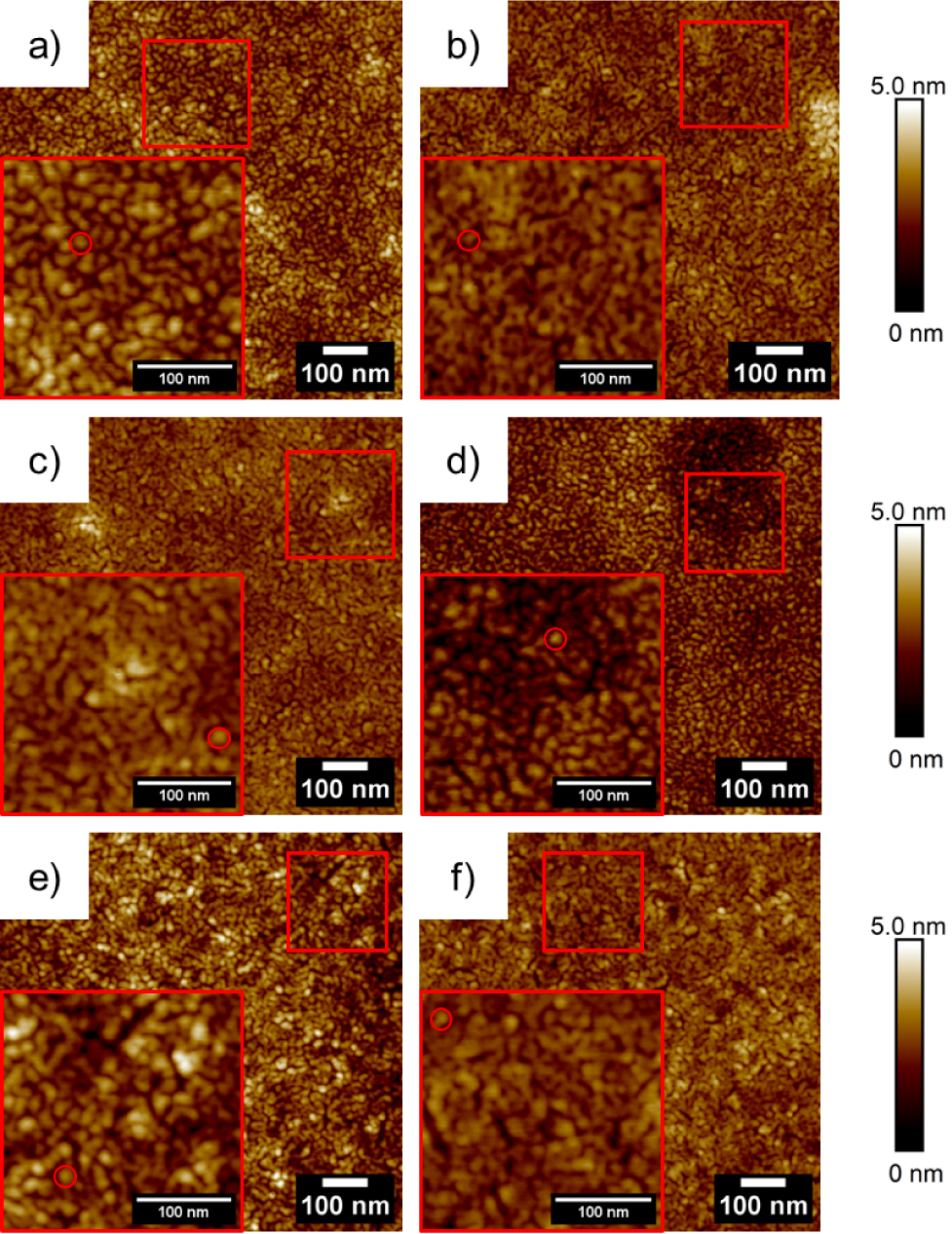
AFM images of (a, c, e) dcMS- and (b, d, f) HiPIMS-deposited gold δ_Au_ = 3 nm on (a, b) Au:PS, (c, d) Au:P4VP, and (e, f) Au:PSS are shown. The red box highlights the magnification of the region of interest. The red circle in the magnified box highlights the isolated Au islands.

From Figure 1a–f at δ_Au_ = 2 nm, it is clear that in both cases, HiPIMS (PS_HiPIMS_, P4VP_HiPIMS_, PSS_HiPIMS_) or dcMS (PS_dcMS_, P4VP_dcMS_, and PSS_dcMS_), Au islands coalesced, forming a branched network, which is visible. The average size of the isolated Au islands is *d*_Au,cluster_ = 4 ± 2 nm. Distinct changes between HIPIMS and dcMS are visible with more Au deposition in the FESEM images, for example, in Figure 1g,j in the case of Au:PS. At δ_Au_ = 4 nm, the surface coverage (Figure S19) of Au:PS_HiPIMS_-deposited Au is 93 ± 1%, which is higher than with Au:PS_dcMS_ deposition being 76 ± 1%. This trend can be further explored with P4VP. Au:P4VP_HiPIMS_ has a coverage of 93 ± 1%, which is higher than that with Au:P4VP_dcMS_ deposition being 77 ± 1%. PSS shows the highest surface coverage after deposition for Au:PSS_HiPIMS_, 96 ± 1%, again being higher than the coverage of Au on the polymer thin films Au:PS and Au:P4VP. Au:PSS_dcMS_ deposition has an intermediate coverage of 84 ± 1%. At δ_Au_ = 8 nm, all polymer films are completely covered with a thin granular gold film (Figure S20). Furthermore, AFM topography images are acquired at δ_Au_ = 3 nm for dcMS- and HiPIMS-deposited Au on Au:PS, Au:P4VP, and Au:PSS, see Figure 2.

In Figure 2a, in the case of dcMS-deposited Au:PS, a network of percolated Au islands is clearly visible, which is not dominantly apparent at δ_Au_ = 2 nm, see Figure 1a. This gives an indication that Au islands start to be in contact with the neighboring Au islands at a thickness of δ_Au,dcMS,PS_ = 3 nm. A similar behavior is also observed for Au:PS_HiPIMS_ (Figure 2b) at a thickness of δ_Au,HiPIMS,PS_ = 3 nm, showing sparsely singular nonpercolated islands, but the majority of the Au islands already percolated into a network. In the case of dcMS and HiPIMS deposition of Au at a thickness of δ_Au_ = 3 nm for Au:P4VP and Au:PSS, similar behavior is observed, as described in the case of PS. The majority of the Au islands are percolated, and small amounts of isolated Au islands are still observable. This trend is observed throughout all of the AFM images in Figure 2. In-situ dcMS and HiPIMS deposition experiments are performed utilizing the GISAXS geometry to exploit its statistical relevance and track the real time evolution of the Au nanostructure up to a thickness of δ_Au_ = 8 nm since an already closed Au layer is observed at that thickness. The aim is to elucidate the mechanism behind the increased coverage comparing HiPIMS and dcMS, similar to the FESEM and AFM measurement; see Figure 1 and Figure 2.

**Figure 3:**
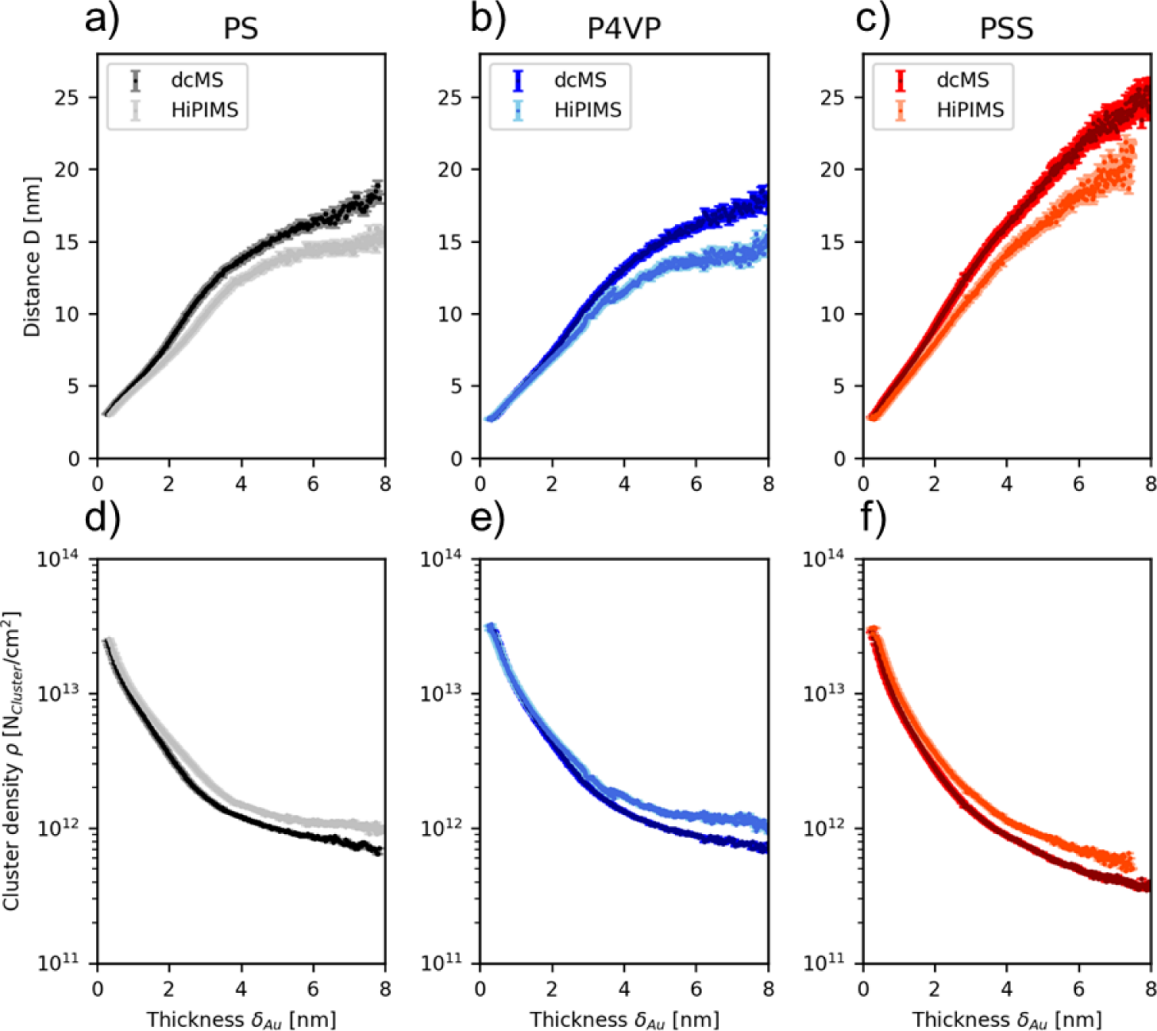
In-situ evolution of the distance of Au islands forming on (a) Au:PS, (b) Au:P4VP, and (c) Au:PSS. In situ evolution of the cluster density of Au islands on (d) Au:PS, (e) Au:P4VP, and (f) Au:PSS.

In Figure 3, the in situ evolution of distance and cluster density of Au deposited on PS, P4VP, and PSS upon dcMS and HiPIMS deposition is presented. The correlation distance (*D*) is extracted from the peak position (*q*_i_), see Figure S24:
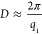
1

In all cases, for 0 nm < δ_Au_ ≤ 0.35 nm, no structure peak is apparent in the in situ GISAXS data, as the very first Au atoms are arriving on the sample surface. First, impinging Au atoms are adsorbed on the polymer surface and Au atoms are diffusing along the substrate surface; stable nuclei are formed when two or more adatoms are being united.^46,47^ Upon continuous deposition, these nuclei grow in the manner of the Volmer–Weber growth, thus growing in height resulting in a low surface coverage, and the probability to form nuclei is reduced, as the new incoming Au atoms grow alongside the preexisting nuclei.^46,47^ The nucleation stage (I) is followed by the lateral growth stage. After a critical cluster density is reached, the apparently formed nuclei and clusters grow in size with new impinging Au atoms during the process.^41,46^ Additionally, the apparent nuclei and clusters are able to diffuse along the surface, resulting in a diffusion-mediated coalescence of clusters into larger structures (stage II).^48^ Both processes result in the increase of the surface coverage of the polymer surface.^46^ The third stage is the coarsening stage in which diffusion of the clusters is greatly reduced; thus, diffusion-mediated coalescence is not the dominant factor anymore. Instead, it will be accommodated by adatoms adsorbing on these clusters supporting the growth in the lateral direction (stage III).^41,46^ This can be observed at δ_Au_ = 2 nm in the FESEM images in Figure 1 showing sparsely isolated Au islands and branched Au islands. During this process, more Au islands branch into a network that can be observed at δ_Au_ = 3 nm in the AFM images in Figure 2. Even upon further deposition, the surface coverage increases due to lateral growth, which can be seen by the FESEM images in Figure 1 at δ_Au_ = 4 nm. The fourth stage is reached when the surface is fully covered. This stage is called vertical growth or grain growth stage, where the film growth occurs only vertically, since the surface is fully covered.^41,46^ Upon continuation of deposition, a polycrystalline grainy structure is obtained, which can be seen in the FESEM images in Figure S23.^46^ In detail, the different stages occur for PS_dcMS_ and PS_HiPIMS_ in the range 0 nm ≤ δ_Au_ ≲ 0.4 nm/0.4 nm (stage I), 0.4 nm/0.4 nm ≤ δ_Au_ ≲ 1.6 nm/1.8 nm (stage II), 1.6 nm/1.8 nm nm ≤ δ_Au_ ≲ 3.7 nm/3.9 nm (stage III), and 3.7 nm/3.9 nm nm ≤ δ_Au_ until the end of the deposition being stage IV. For P4VP_dcMS_ and P4VP_HiPIMS_, the ranges were 0 nm ≤ δ_Au_ ≲ 0.4 nm/0.4 nm (stage I), and 0.4 nm/0.4 nm ≤ δ_Au_ ≲ 1.8 nm/1.8 nm (stage II), 1.8 nm/1.8 nm ≤ δ_Au_ ≲ 3.7 nm/3.9 nm (stage III), and 3.9 nm/4.2 nm ≤ δ_Au_ until the end of the deposition being stage IV. Finally, for PSS_dcMS_ and PSS_HiPIMS_, the ranges were 0 nm ≤ δ_Au_ ≲ 0.4 nm/0.4 nm (stage I), 0.4 nm/0.4 nm ≤ δ_Au_ ≲ 1.6 nm/1.7 nm (stage II), 1.6 nm/1.7 nm ≤ δ_Au_ ≲ 3.7 nm/3.9 nm (stage III), and 3.7 nm/4.0 nm ≤ δ_Au_ until the end of the deposition being stage IV. Clearly, strong deviations in cluster sizes and distance start to occur in stage II for dcMS and HiPIMS. This is attributed to the increased initial cluster density, see Figure 3, due to the difference in the kinetic energy distribution of the ionized fraction of the impinging atoms. The same fundamental behavior is expected on PS, P4VP, and PSS due to the similarity in the polymer structure. Here, it is important to note the difference during Au deposition between dcMS and HiPIMS on PS, P4VP, and PSS. In Figure 3a, the in situ structure evolution of Au:PS_dcMS_ and Au:PS_HiPIMS_ is shown, being similar in the range δ_Au_ = 0.35 nm up to δ_Au_ = 1.8 nm. Beyond δ_Au_ = 1.8 nm, it is obvious that Au:PS_dcMS_ has a larger average distance during the in situ evolution compared to Au:PS_HiPIMS_. This trend continues until the end of the in situ sequence probed in the present study. In Figure 3b, the in situ structure evolution of Au:P4VP_dcMS_ and Au:P4VP_HiPIMS_ is shown, and within errors from δ_Au_ = 0.35 nm up to δ_Au_ = 2.4 nm, the average distances are similar. Au:P4VP_dcMS_ again has a larger average correlation distance compared to Au:P4VP_HiPIMS_ toward the end of the in situ sequence. In Figure 3c, the in situ sequences of Au:PSS_dcMS_ and Au:PSS_HiPIMS_ are displayed. Here, from δ_Au_ = 0.35 nm up to δ_Au_ = 2.4 nm, Au:PSS_dcMS_ and Au:PSS_HiPIMS_ islands have a similar distance. Upon δ_Au_ = 2.4 nm, Au:PSS_dcMS_ has an average higher distance than Au:PSS_HiPIMS_ similar to our findings for Au:PS and Au:P4VP. Additionally, after δ_Au_ = 8 nm for PS_HiPIMS_, P4VP_dcMS_, P4VP_HiPIMS_, and PSS_dcMS_, no structure peak of Au is being observed anymore in this particular GISAXS geometry. For PS_dcMS_ and PSS_HiPIMS_, no clear structure peak is observed already at δ_Au_ = 7.9 nm and δ_Au_ = 7.6 nm, respectively. Hence, the abscissa in the corresponding figures ends at δ_Au_ = 8 nm and not at the final deposited thickness. With no structure peak being present, it is not possible to track the Au growth between δ_Au_ = 8 nm and the final targeted deposited thickness around 12 nm for each sample (see Figure S4) in this GISAXS geometry. The reason is that either the average correlated distance sizes are too large to be resolved at this particular chosen distance for the GISAXS measurement or no average structure is present on the sample surface after this deposited thickness. In case larger distances are present on the sample surface after δ_Au_ = 8 nm, a larger sample-to-detector distance in the GISAXS geometry is required to resolve these larger structures. In order to further quantify the Au islands’ layer morphology, it is derived using the following relation:^38^
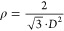
2

It is clearly seen that the number of islands for PS_dcMS_ and PS_HiPIMS_ (Figure 3d) decreases during the deposition. This behavior is due to the coalescence of Au islands. Yet, it is worth noting that the number of Au islands is higher for PS_HiPIMS_ than for PS_dcMS_ until the end of the deposition process. This observation explains the increased coverage of PS_HiPIMS_ compared to PS_dcMS_ seen in Figure 1g,j) since a larger number of islands is apparent during the structure evolution. In the case of P4VP (Figure 3e), the same trend is observable. For δ_Au_ ≥ 2 nm, Au:P4VP_HiPIMS_ shows a larger number of islands during deposition compared to Au:P4VP_dcMS_, which again explains the increase in coverage for HiPIMS in Figure 1h,k. The same trend is observable for PSS (Figure 3f). It is clearly seen that Au:PSS_HiPIMS_ has an increased number of islands compared to Au:PSS_dcMS_ during the deposition, which supports the observation of the increase in coverage in Figure 1 i,l. From these results, it is possible to deduce that under HiPIMS conditions, an increased amount of nucleation sites are formed due to defect formation induced by an increase of the average kinetic energy of the ionized Au fraction of HiPIMS 9.7 ± 0.1 eV compared to dcMS 3.9 ± 0.1 eV.^49^ Reck et al. shows by studying the effect of dcMS and HiPIMS on silicon substrate and polystyrene substrate with Ag deposition that the probability of creating surface defects with fast metal ions is enhanced.^50^ The increased average kinetic energy results in an increased number of Au islands with a smaller size and correspondingly in a smaller correlation distance during sputter deposition. This finding is supported by the FESEM images in Figure 1g–l, showing that the coverage by HiPIMS deposition is increased due to the appearance of an increased number of smaller islands (compared to dcMS). This in turn manifests itself in the higher cluster density for HiPIMS. In previous investigations, the Au deposition on PS was investigated.^38^ In order to extract information on the radius of the Au islands, the cluster shape was approximated as being hemispherical.^38^ By taking the ratio of the cluster diameter to the average distance, we obtained information on the percolation threshold. The percolation threshold defines the nominal layer thickness, the moment in which islands are in direct contact with the neighboring islands, resulting in conductive metallic behavior of the nanogranular film. This geometric hemispherical model considers the structure peak position *q*_i_, the amount of Au in one cluster, and the cluster shape (hemisphere). Thus, the radius is thus related to the deposited material and the cluster distance via:^38^
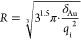
3

It is noteworthy to mention that the derived radius is model- and shape-dependent, as outlined below, see eq 5 and ref (52).

**Figure 4:**
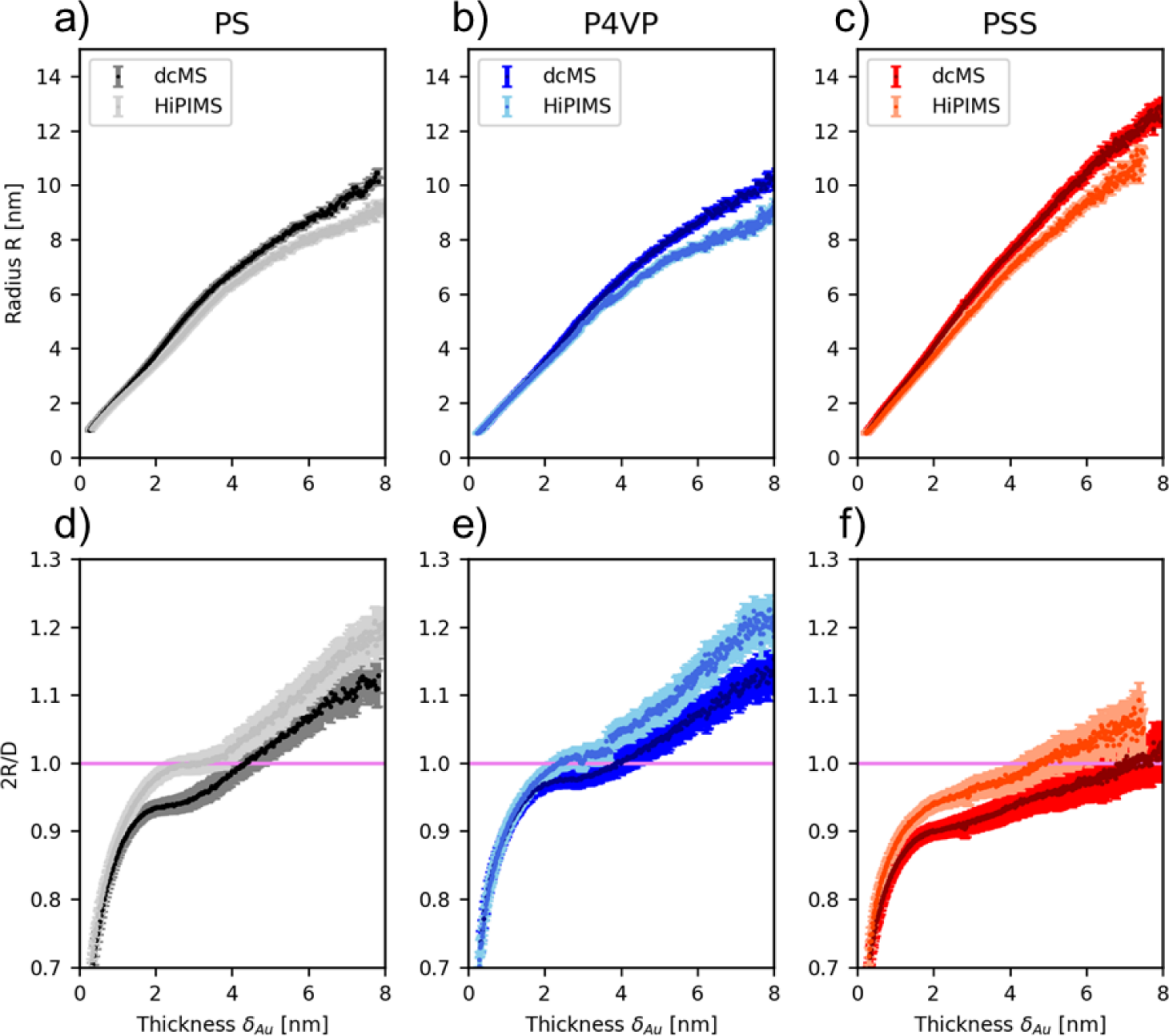
In-situ evolution of the radius *R* derived from the hemispherical geometrical model for (a) Au:PS, (b) Au:P4VP, and (c) Au:PSS. In-situ evolution of the ratio two times the radius (2*R* = cluster diameter) and the correlation distance *D* to determine the percolation threshold for (d) Au:PS, (e) Au:P4VP, and (f) Au:PSS. The pink line at 2*R*/*D* = 1.0 in (d–f) denotes the percolation threshold.

In Figure 4, the in situ evolution of the cluster sizes and the ratio 2*R*/*D* of PS, P4VP, and PSS is depicted as extracted from the geometrical model. The evolution of radii *R* of Au islands on Au:PS_dcMS_ and Au:PS_HiPIMS_ during deposition shows that the Au islands on Au:PS_HiPIMS_ have a smaller radius than the Au islands on Au:PS_dcMS_ (Figure 4a) above δ_Au_ ≥ 1.8 nm. This trend holds during full in situ evolution. Moreover, in Figure 4b, in case of Au:P4VP, it is observable only after δ_Au_ = 2.4 nm that Au:P4VP_HiPIMS_ islands have a smaller radius during the subsequent evolution than Au:P4VP_dcMS_. This behavior continued until the end of the deposition process. Furthermore, in Figure 4c, the radius evolution during in situ deposition of Au:PSS_HiPIMS_ and Au:PSS_dcMS_ is shown. Au:PSS_HiPIMS_ has on average a smaller radius than Au:PSS_dcMS_. Thus, Au cluster sizes in all polymers show the same trend during deposition, which is the result of the average increased kinetic energy of the ionized fraction, as mentioned above. Additionally, using the geometrical model, it is possible to calculate the ratio of the diameter (2*R*) and the distance between the islands. When this ratio 2*R*/*D* is equal to 1, this implies that on average throughout the sample, every cluster is touching its neighbor and a conductive path is formed. In Figure 4d, 2*R*/*D* for Au:PS_dcMS_ has a percolation threshold of δ_Au,Percolation_ = 3.9 ± 0.5 nm, while Au:PS_HiPIMS_ has a percolation threshold of δ_Au,Percolation_ = 3.0 ± 0.7 nm, which is in agreement with our previous investigation.^38^ In Figure 4e, it is visible that Au:P4VP_dcMS_ percolates at δ_Au,percolation_ = 4.0 ± 0.5 nm, and in the case of Au:P4VP_HiPIMS_, it percolates at δ_Au,percolation_ = 2.4 ± 0.3 nm. Moreover, Figure 4f shows that Au:PSS_dcMS_ has a percolation threshold of δ_Au,percolation_ = 7.0 ± 1.0 nm, and in the case of HiPIMS deposition, Au:PSS_HiPIMS_ has a percolation threshold of δ_Au,percolation_ = 4.7 ± 0.6 nm.

**Figure 5:**
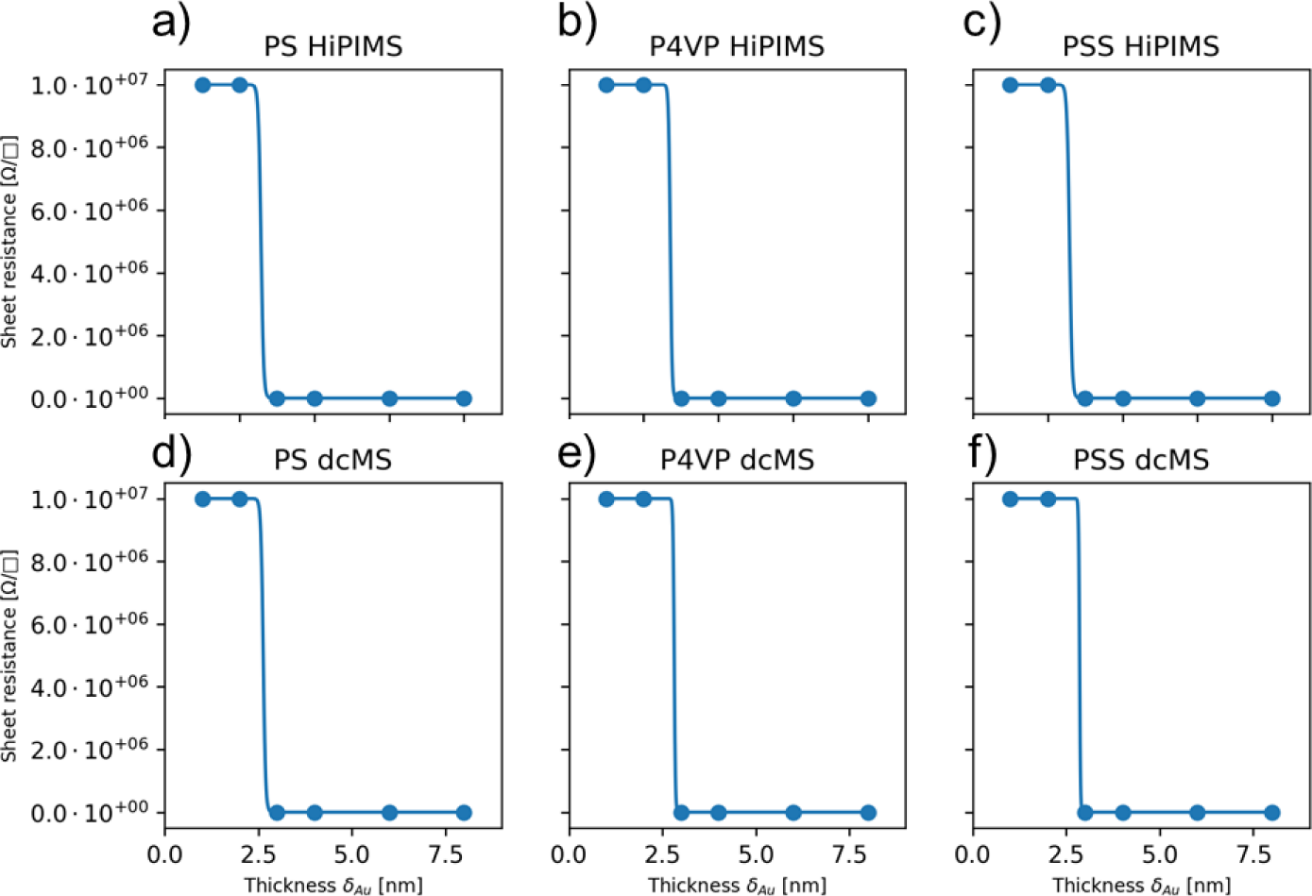
4-point-probe measurements with the corresponding sigmoidal Boltzmann fit for (a) Au:PS_HiPIMS_, (b) Au:P4VP_HiPIMS_, (c) Au:PSS_HiPIMS_, (d) Au:PS_dcMS_, (e) Au:P4VP_dcMS_, and (f) Au:PSS_dcMS_ with a deposited thickness being δ_Au_ = 1, 2, 3, 4, 6, and 8 nm.

In Figure 5, four-point-probe measurements of ex-situ deposited PS, P4VP, and PSS are shown with the corresponding sigmoidal Boltzmann fit for the determination of the percolation threshold.^51^ At δ_Au_ = 1 nm and 2 nm, no conductivity was measured and the sheet resistance was set to 10 MΩ due to the upper limit of the 4-point probe to measure resistance. Figure 5a,d shows the resistivity measurements for Au:PS_dcMS_ and Au:PS_HiPIMS_ with the corresponding fits. The percolation thresholds are δ_Au,percolation_ = 2.6 ± 0.1 nm for Au:PS_dcMS_ and δ_Au,percolation_ = 2.6 ± 0.1 nm for Au:PS_HiPIMS_. The percolation threshold for Au:P4VP_dcMS_ and Au:P4VP_HiPIMS_ (Figure 5b) is δ_Au,percolation_ = 2.8 ± 0.2 nm for Au:P4VP_dcMS_ for P4VP_HiPIMS_ is δ_Au,percolation_ = 2.7 ± 0.1 nm, which is earlier than Au:PV4P_dcMS_. A similar behavior is observed in Figure 5c for PSS (Au:PSS_dcMS_ δ_Au,percolation_ = 2.9 ± 0.1 nm vs Au:PSS_HiPIMS_ δ_Au,percolation_ = 2.6 ± 0.1 nm). In Table 1, the results regarding the percolation threshold extracted by GISAXS utilizing the hemispherical model and the 4-point-probe measurements are summed up. The comparison shows that the agreement of Au:PS_HiPIMS_ and Au:P4VP_HiPIMS_ fits well with four-point-probe measurements and the hemispherical model. In the case of Au:PS_dcMS_ and Au:P4VP_dcMS_, there is a slight discrepancy apparently of roughly ∼1 nm matching the percolation threshold. This discrepancy occurs due to the simplicity of the hemispherical model only considering the monodisperse size of Au islands during the growth on a triangular arrangement. Particularly, for Au:PSS_dcMS_ and Au:PSS_HiPIMS_, the discrepancy is enhanced. One reason is, as described, that the hemispherical model considers only monodisperse Au islands. On the other hand, it can be seen in the FESEM images in Figure 1 that the surface coverage of Au on PSS is larger under dcMS and HiPIMS conditions compared to PS and P4VP. As it is extracted from the GISAXS data, Au growing on PSS either in dcMS or HiPIMS conditions leads to larger structures than PS and P4VP, which can be seen in Figure S23. Particularly, the cluster density is lower for Au growing on PSS compared with PS and P4VP, which can be seen in Figure S24. We expect that the higher surface energy for PSS leads to an improved wetting behavior and that faster larger structures are grown. Due to the faster growth, the structures are more oblate compared to the simple hemispherical model. In a previous in situ investigation of Cu growth on PS-*b*-PEO, it could be seen that the Cu islands deviate strongly from the hemispherical shape. They grow more elongated in the height being described by the hemispherical elongation factor with *H* being the height and *R* being the radius of the Cu islands:^52^
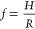
4
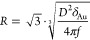
5

**Table 1:** Summary of the Percolation Threshold Extracted by 4-Point-Probe Measurements and GISAXS Data Utilizing the Hemispherical Model

Material	Percolation threshold 4-point-probe (nm)	Percolation threshold hemispherical model (nm)
PS dcMS	2.6 ± 0.1	3.9 ± 0.5
PS HiPIMS	2.6 ± 0.1	3.0 ± 0.7
P4VP dcMS	2.8 ± 0.1	4.0 ± 0.5
P4VP HiPIMS	2.7 ± 0.1	2.4 ± 0.3
PSS dcMS	2.9 ± 0.1	7.0 ± 1.0
PSS HiPIMS	2.6 ± 0.1	4.7 ± 0.6

Schaper et al. observed in this case that the Cu islands had a hemispherical elongation factor *f* = 2.75 due to the strong Cu–Cu interaction and surface containing oxygen due to the diblock copolymer PS-*b*-PEO.^52^ In the case of the hemispherical model, the elongation factor is *f* = 1. We expect for PSS that the hemispherical elongation factor *f* is lower than 1 due to the increased surface coverage being observed in Figure 1 for PSS at δ_Au_ = 4 nm. Using the hemispherical elongation factor of *f* = 0.9 for PSS_dcMS_ and PSS_HiPIMS_, results are matching with the percolation threshold for PSS_HiPIMS_ being at δ_Au_ = 2.6 ± 0.6 nm. In the case of PSS_dcMS_, the percolation threshold would result in δ_Au_ = 3.9 ± 1.0 nm. Thus, the introduction of a microscopy-based elongation factor reduces the difference between the 4-point-probe and model-based extraction of percolation threshold in Table 1 for PSS.

**Figure 6:**
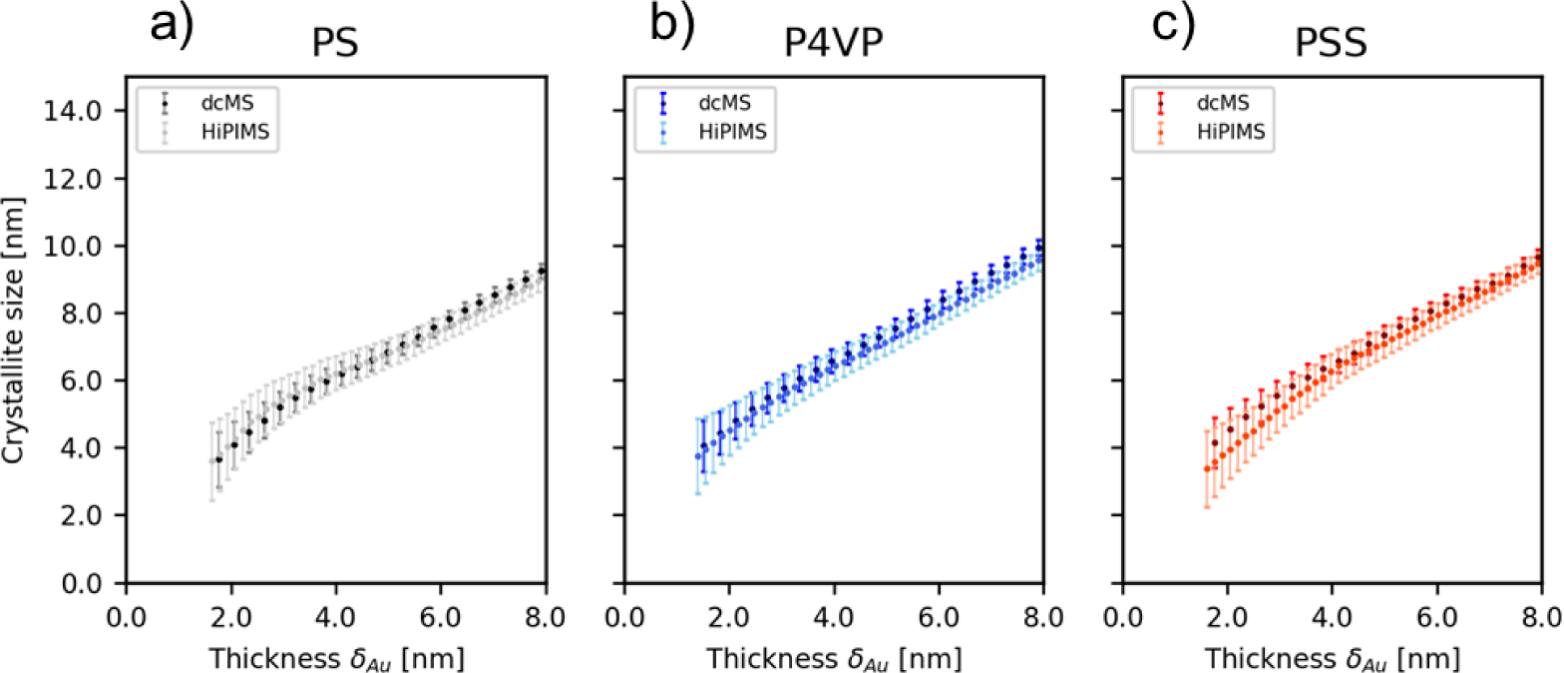
In-situ GIWAXS evolution of the crystallite size of (a) Au:PS, (b) Au:P4VP, and (c) Au:PSS.

For completeness, the in situ GIWAXS evolution during deposition is displayed in Figure 6. GIWAXS is used in situ during the sputter deposition to detect the crystallite size evolution. The in situ crystallite size evolution for Au on PS, P4VP, and PSS deposited with dcMS and HiPIMS condition is displayed in Figure 6. Due to experimental restrictions of the in situ GIWAXS geometry for the simultaneous combination with in-situ GISAXS and sputter deposition, only a fraction of the necessary angular range is measured for strain analysis. Thus, a complete strain analysis representing the sample is hence omitted.^53,54^ It has to be noted that the strain analysis can influence the crystallite size by the Scherrer analysis. The crystallite size was extracted following Figure S22 using the Scherrer equation with *K* = 0.9 and coressponds to the <111> reflex of Au.^55,56^ In the case of Au:PS_dcMS_, the first noticeable Au crystallites can be detected at around δ_Au,dcMS_ = 1.8 nm with the size being 3.7 ± 0.8 nm (Figure 6a). Upon further deposition, the crystallite size increases to 9.2 ± 0.2 nm at the final deposited thickness δ_Au,dcMS_ = 8 nm. In the case of Au:PS_HiPIMS_, the same trend can be observed. First noticeable crystallites start to appear at around δ_Au_ = 1.7 nm, with their size being 3.8 ± 1.0 nm. With the continuation of the deposition process, the crystallite size increases up to 8.9 ± 0.3 nm at a final deposited thickness of δ_Au_ = 8 nm. In the case of Au:P4VP_dcMS_ and Au:P4VP_HiPIMS_, the same trend is observed when Au was deposited on P4VP_dcMS_. First noticeable crystallites start to form at δ_Au,dcMS_ = 1.8 nm with the average crystallite size being 4.4 ± 0.6 nm, and at δ_Au,dcMS_ = 8 nm, the crystallite size has increased to 9.9 ± 0.2 nm. This behavior is in a similar range as Au:P4VP_HiPIMS_, which shows first noticeable crystallites in the size of 3.7 ± 1.1 nm at δ_Au,HiPIMS_ = 1.4 nm and a final crystallite size of 9.5 ± 0.3 nm at the final deposited thickness δ_Au,HiPIMS_ = 8 nm. In the case of Au:PSS_dcMS_ in Figure 6c, the first noticeable crystallites occur at δ_Au,dcMS_ = 1.8 nm with a size of 4.1 ± 0.7 nm, which increases during deposition to 9.7 ± 0.2 nm. For Au:PSS_HiPIMS_ noticeable crystallites start to form at δ_Au,HiPIMS_ = 1.6 nm with the average crystallite size being 3.7 ± 1.1 nm and increase to 9.5 ± 0.3 nm at δ_Au,HiPIMS_ = 8 nm. This observation shows that by keeping the dcMS and HiPIMS conditions similar, the crystallite size is the same during deposition. Moreover, in all cases, the crystallite size increased upon continuous Au deposition. Additionally, independent from the polymer, it can be clearly seen that there is no difference in the crystallite size for the polymers PS, P4VP, and PSS for both dcMS and HiPIMS conditions. Only their radius, distance, and cluster density differ. Particularly, differences during the in situ deposition are observed within the polymers in terms of distance (Figure S23), cluster density (Figure S24), and the radius (Figure S25). In Table 2 at various Au thicknesses, the distance, radius, crystallite size, and sheet resistance are summarized. In the case of the dcMS deposition (Figures S23 and S25) environment, PS has, with its phenyl functional groups, bigger distances and radius than P4VP, with its pyridine functional group, up to δ_Au,dcMS_ = 4.1 nm. After that threshold value, the distance and radius are similar. Additionally, until δ_Au,dcMS_ = 4.1 nm, P4VP has a higher cluster density than PS, which after that is similar. In contrast, PSS has throughout the in situ deposition a bigger distance and radius than PS and P4VP, which is induced due to the sulfonic acid functional group and has the smallest amount of cluster density during deposition. In case of HiPIMS deposition, environment PSS has still the biggest radius and distance throughout the deposition and the lowest cluster density. In case of PS and P4VP, both have the same radius and distance size, which originates due to the increased kinetic energy distribution of the ionized fraction during deposition, showing a reduced influence of the pyridine functional group of P4VP toward the phenyl functional group of PS. Löhrer et al. showed in a previous study already that the growth of Au is influenced by an addition of a benzodithiophene functional group resulting in a change of distance and radius during deposition.^28^

**Table 2:** Summary of Distance, Radius, Crystallite Size, and Sheet Resistance of Au Under dcMS and HiPIMS Conditions for PS, P4VP, and PSS^a^

		Distance [nm]		Radius [nm]		Crystallite size [nm]		Sheet resistance [Ω/□]	
Material	Thickness (δ_Au_) [nm]	dcMS	HiPIMS	dcMS	HiPIMS	dcMS	HiPIMS	dcMS	HiPIMS
PS	1	5.2 ± 0.1	4.8 ± 0.1	2.2 ± 0.1	2.1 ± 0.1	-	-	-*	-*
	2	8.2 ± 0.1	7.1 ± 0.1	3.9 ± 0.1	3.5 ± 0.1	4.1 ± 0.7	4.3 ± 0.9	-*	-*
	3	11.7 ± 0.1	9.9 ± 0.1	5.5 ± 0.1	5.0 ± 0.1	5.2 ± 0.5	5.4 ± 0.7	93.8 ± 0.1	53.3 ± 0.1
	4	13.8 ± 0.2	12.4 ± 0.1	6.8 ± 0.1	6.3 ± 0.1	6.2 ± 0.3	6.2 ± 0.5	31.5 ± 0.1	25.5± 0.1
	6	16.3 ± 0.3	14.4 ± 0.1	8.7 ± 0.1	8.1 ± 0.1	7.6 ± 0.3	7.4 ± 0.4	16.2 ± 0.1	14.0 ± 0.1
	8	-	15.8 ± 0.3	-	9.4 ± 0.1	9.2 ± 0.2	8.9 ± 0.3	9.9 ± 0.1	9.3 ± 0.1
P4VP	1	4.5 ± 0.1	4.5 ± 0.1	2.0 ± 0.1	2.0 ± 0.1	-	-	-*	-*
	2	7.3 ± 0.1	6.9 ± 0.1	3.5 ± 0.1	3.4 ± 0.1	4.8 ± 0.5	4.5 ± 0.7	-*	-*
	3	10.6 ± 0.1	9.9 ± 0.1	5.2 ± 0.1	4.9 ± 0.1	5.8 ± 0.4	5.5 ± 0.5	126.5 ± 0.1	55.6 ± 0.1
	4	13.2 ± 0.2	11.5 ± 0.1	6.6 ± 0.1	6.0 ± 0.1	6.6 ± 0.3	6.4 ± 0.4	37.6 ± 0.1	24.1 ± 0.1
	6	16.0 ± 0.3	13.7 ± 0.2	8.6 ± 0.1	7.7 ± 0.1	8.4 ± 0.2	8.0 ± 0.3	16.1 ± 0.1	12.8 ± 0.1
	8	17.2 ± 0.3	15.8 ± 0.3	10.0 ± 0.1	9.4 ± 0.1	9.9 ± 0.2	9.5 ± 0.3	10.9 ± 0.1	8.7 ± 0.1
PSS	1	5.2 ± 0.1	4.9 ± 0.1	2.3 ± 0.1	2.1 ± 0.1	-	-	-*	-*
	2	8.2 ± 0.1	4.8 ± 0.1	4.1 ± 0.1	3.7 ± 0.1	4.6 ± 0.6	4.0 ± 0.9	-*	-*
	3	11.7 ± 0.1	7.1 ± 0.1	5.9 ± 0.1	5.4 ± 0.1	5.5 ± 0.4	5.2 ± 0.6	120.4 ± 0.1	72.6 ± 0.1
	4	13.8 ± 0.2	9.9 ± 0.1	7.5 ± 0.1	6.9 ± 0.1	6.6 ± 0.3	6.3 ± 0.5	49.2 ± 0.1	28.1 ± 0.1
	6	16.3 ± 0.3	12.4 ± 0.1	10.5 ± 0.2	9.4 ± 0.1	8.3 ± 0.3	7.9 ± 0.4	16.1 ± 0.1	12.4 ± 0.1
	8	-	14.4 ± 0.1	12.9 ± 0.2	-	9.7 ± 0.2	9.5 ± 0.3	11.5 ± 0.1	8.6 ± 0.1

a *: no conductivity was measured and the sheet resistance was set to 10 MΩ, which is the upper limit of the 4-point-probe range to measure the sheet resistance.

## Conclusion

Using a broad portfolio of real space and reciprocal space imaging methods, we present the nanoscale understanding of the influence of the atom kinetic energies and ionized fraction on the polymer–metal interface induced by two very important physical vapor deposition methods. This study about the dcMS and HiPIMS deposition of Au on thin PS, P4VP, and PSS films shows for early stages at δ_Au_ = 2 nm that only well separated Au islands are apparent. At δ_Au_ = 4 nm, HiPIMS deposition greatly increases the coverage compared to the dcMS. GISAXS reveals that an increase of the cluster density is responsible for an increase of coverage, which occurs due to an increase of nucleation points during the deposition. Furthermore, four-point-probe measurements reveal that HiPIMS-deposited Au has an earlier percolation threshold only for PSS. In-situ GISAXS investigation using the hemispherical model reveals that under the HiPIMS condition, the percolation threshold is earlier than dcMS for all polymeric templates. Additionally, it can be extracted that HiPIMS-deposited Au islands have a smaller average radius compared to dcMS-deposited Au islands. Furthermore, GIWAXS measurements reveal that with the chosen dcMS and HiPIMS parameters, the crystallite sizes remain similar throughout the entire deposition being independent from the chosen polymeric templates. These results provide a profound understanding of the formation of thin gold electrodes on polymers. It can be summed up that these results show that with HiPIMS deposition an increased coverage is achieved which is important for applications in the field of organic electronics. Future work fill focus on the effect of pulse duration and frequency on the growth behavior and microstructure.

## Supporting Information Available

Skeletal structure of the polymers; XRR measurements; SLD profiles; FESEM measurements; GISAXS and GIWAXS detector images; surface coverage analysis with FESEM; GISAXS and GIWAXS scattering profiles with the corresponding fit; comparison of in situ analysis; comparison of FESEM- and GISAXS-derived cluster density; sheet resistance measurement (PDF)

## References

[ref1] Zhang M.; Liu Y.; Du Y.; Liu H. Tuning Topology and Scaffolding Units in Nanoporous Polymeric Materials for Efficient Iodine Adsorption and Detection. ACS Appl. Nano Mater. 2023, 6, 13874 10.1021/acsanm.3c00723.

[ref2] Ojha M.; Pal R. K.; Deepa M. Selenium/g-C 3 N 4 with a Solid Li 4 Ti 5 O 12 Blocking Layer for Selective Li + Ion Diffusion in Long-Lived Li–Se Batteries. ACS Appl. Nano Mater. 2023, 6 (15), 13912–13925. 10.1021/acsanm.3c01580.

[ref3] Kumar Bera A.; Singh S.; Shahid Jamal M.; Hussain Z.; Reddy V. R.; Kumar D. Growth and In-Situ Characterization of Magnetic Anisotropy of Epitaxial Fe Thin Film on Ion-Sculpted Ag (001) Substrate. J. Magn. Magn. Mater. 2022, 544, 168679 10.1016/j.jmmm.2021.168679.

[ref4] Ren H.-T.; Cai C.-C.; Cao W.-B.; Li D.-S.; Li T.-T.; Lou C.-W.; Lin J.-H. Superhydrophobic TiN-Coated Cotton Fabrics with Nanoscale Roughness and Photothermal Self-Healing Properties for Effective Oil–Water Separation. ACS Appl. Nano Mater. 2023, 6 (13), 11925–11933. 10.1021/acsanm.3c01763.

[ref5] Morisue M.; Hoshino Y.; Shimizu M.; Tomita S.; Sasaki S.; Sakurai S.; Hikima T.; Kawamura A.; Kohri M.; Matsui J.; Yamao T. A Metal-Lustrous Porphyrin Foil. Chem. Commun. 2017, 53 (77), 10703–10706. 10.1039/C7CC06159E. 28913537

[ref6] Chen A. X.; Lau H. Y.; Teo J. Y.; Wang Y.; Choong D. Z. Y.; Wang Y.; Luo H.-K.; Yang Y. Y.; Li N. Water-Mediated In Situ Fabrication of CuI Nanoparticles on Flexible Cotton Fabrics as a Sustainable and Skin-Compatible Coating with Broad-Spectrum Antimicrobial Efficacy. ACS Appl. Nano Mater. 2023, 6 (14), 13238–13249. 10.1021/acsanm.3c01961.

[ref7] Buffet A.; Abul Kashem M. M.; Schlage K.; Couet S.; Röhlsberger R.; Rothkirch A.; Herzog G.; Metwalli E.; Meier R.; Kaune G.; Rawolle M.; Müller-Buschbaum P.; Gehrke R.; Roth S. V. Time-Resolved Ultrathin Cobalt Film Growth on a Colloidal Polymer Template. Langmuir 2011, 27 (1), 343–346. 10.1021/la102900v. 21117670

[ref8] Ziefuss A. R.; Steenbock T.; Benner D.; Plech A.; Göttlicher J.; Teubner M.; Grimm-Lebsanft B.; Rehbock C.; Comby-Zerbino C.; Antoine R.; et al. Photoluminescence of Fully Inorganic Colloidal Gold Nanocluster and Their Manipulation Using Surface Charge Effects. Adv. Mater. 2021, 33 (31), 2101549 10.1002/adma.202101549. 34165866 PMC11469328

[ref9] Wang Y.; Chen J.; Zhong Y.; Jeong S.; Li R.; Ye X. Structural Diversity in Dimension-Controlled Assemblies of Tetrahedral Gold Nanocrystals. J. Am. Chem. Soc. 2022, 144 (30), 13538–13546. 10.1021/jacs.2c03196. 35863043

[ref10] Sousa G. P.; de Barros A.; Shimizu F. M.; Sigoli F. A.; Mazali I. O. Plasmonic Photocatalysis Driven by Indirect Gold Excitation Via Upconversion Nanoparticle Emission Monitored In Situ by Surface-Enhanced Raman Spectroscopy. ACS Appl. Nano Mater. 2023, 6, 9206 10.1021/acsanm.3c00704.

[ref11] Amarandei G.; O’Dwyer C.; Arshak A.; Corcoran D. Fractal Patterning of Nanoparticles on Polymer Films and Their SERS Capabilities. ACS Appl. Mater. Interfaces 2013, 5 (17), 8655–8662. 10.1021/am402285e. 23980932

[ref12] Metwalli E.; Couet S.; Schlage K.; Röhlsberger R.; Körstgens V.; Ruderer M.; Wang W.; Kaune G.; Roth S. V.; Müller-Buschbaum P. In Situ GISAXS Investigation of Gold Sputtering onto a Polymer Template. Langmuir 2008, 24 (8), 4265–4272. 10.1021/la7038587. 18302441

[ref13] Soria E.; Gomez-Rodriguez P.; Tromas C.; Camelio S.; Babonneau D.; Serna R.; Gonzalo J.; Toudert J. Self-Assembled, 10 Nm-Tailored, Near Infrared Plasmonic Metasurface Acting as Broadband Omnidirectional Polarizing Mirror. Adv. Opt. Mater 2020, 8 (21), 2000321 10.1002/adom.202000321.

[ref14] Zheng T.; Kwon H.; Faraon A. Nanoelectromechanical Tuning of High- Q Slot Metasurfaces. Nano Lett. 2023, 23 (12), 5588–5594. 10.1021/acs.nanolett.3c00999. 37306317 PMC10311603

[ref15] Walter H.; Leitner A. Role of Granular Structure in Metal Layers on the Optical Properties of Absorbing Mirrors. Opt. Eng. 2006, 45 (10), 103801 10.1117/1.2363167.

[ref16] Frank M.; Bulut Y.; Czympiel L.; Weißing R.; Nahrstedt V.; Wilhelm M.; Grosch M.; Raauf A.; Verma A.; Fischer T.; et al. Piezo-Enhanced Activation of Dinitrogen for Room Temperature Production of Ammonia. Nanotechnology 2021, 32 (46), 465601 10.1088/1361-6528/ac1a96. 34348241

[ref17] Cai X.; Li G.; Hu W.; Zhu Y. Catalytic Conversion of CO2over Atomically Precise Gold-Based Cluster Catalysts. ACS Catal. 2022, 12 (17), 10638–10653. 10.1021/acscatal.2c02595.

[ref18] Sankar M.; He Q.; Engel R. V.; Sainna M. A.; Logsdail A. J.; Roldan A.; Willock D. J.; Agarwal N.; Kiely C. J.; Hutchings G. J. Role of the Support in Gold-Containing Nanoparticles as Heterogeneous Catalysts. Chem. Rev. 2020, 120 (8), 3890–3938. 10.1021/acs.chemrev.9b00662. 32223178 PMC7181275

[ref19] Langer N.; LeGrand M.; Kedem O. Cationic Polymer Coating Increases the Catalytic Activity of Gold Nanoparticles toward Anionic Substrates. ACS Appl. Mater. Interfaces 2023, 15 (24), 29160–29169. 10.1021/acsami.3c04087. 37289992

[ref20] Kang T.; Zhu J.; Luo X.; Jia W.; Wu P.; Cai C. Controlled Self-Assembly of a Close-Packed Gold Octahedra Array for SERS Sensing Exosomal MicroRNAs. Anal. Chem. 2021, 93 (4), 2519–2526. 10.1021/acs.analchem.0c04561. 33404216

[ref21] Meyer S. M.; Murphy C. J. Anisotropic Silica Coating on Gold Nanorods Boosts Their Potential as SERS Sensors. Nanoscale 2022, 14 (13), 5214–5226. 10.1039/D1NR07918B. 35315863

[ref22] Grys D.-B.; Niihori M.; Arul R.; Sibug-Torres S. M.; Wyatt E. W.; de Nijs B.; Baumberg J. J. Controlling Atomic-Scale Restructuring and Cleaning of Gold Nanogap Multilayers for Surface-Enhanced Raman Scattering Sensing. ACS Sens. 2023, 8 (7), 2879–2888. 10.1021/acssensors.3c00967. 37411019 PMC10391707

[ref23] Guo P.; Zhu H.; Zhao W.; Liu C.; Zhu L.; Ye Q.; Jia N.; Wang H.; Zhang X.; Huang W.; Vinokurov V. A.; Ivanov E.; Shchukin D.; Harvey D.; Ulloa J. M.; Hierro A.; Wang H. Interfacial Embedding of Laser-Manufactured Fluorinated Gold Clusters Enabling Stable Perovskite Solar Cells with Efficiency Over 24%. Adv. Mater. 2021, 33 (36), 1–11. 10.1002/adma.202101590. 34302406

[ref24] Notarianni M.; Vernon K.; Chou A.; Aljada M.; Liu J.; Motta N. Plasmonic Effect of Gold Nanoparticles in Organic Solar Cells. Sol. Energy 2014, 106, 23–37. 10.1016/j.solener.2013.09.026.

[ref25] Domanski K.; Correa-Baena J. P.; Mine N.; Nazeeruddin M. K.; Abate A.; Saliba M.; Tress W.; Hagfeldt A.; Grätzel M. Not All That Glitters Is Gold: Metal-Migration-Induced Degradation in Perovskite Solar Cells. ACS Nano 2016, 10 (6), 6306–6314. 10.1021/acsnano.6b02613. 27187798

[ref26] Cao S.; Yu D.; Lin Y.; Zhang C.; Lu L.; Yin M.; Zhu X.; Chen X.; Li D. Light Propagation in Flexible Thin-Film Amorphous Silicon Solar Cells with Nanotextured Metal Back Reflectors. ACS Appl. Mater. Interfaces 2020, 12 (23), 26184–26192. 10.1021/acsami.0c05330. 32392028

[ref27] Goyal A.; Koper M. T. M. The Interrelated Effect of Cations and Electrolyte PH on the Hydrogen Evolution Reaction on Gold Electrodes in Alkaline Media. Angew. Chem., Int. Ed. 2021, 60 (24), 13452–13462. 10.1002/anie.202102803. PMC8252582 33769646

[ref28] Löhrer F. C.; Körstgens V.; Semino G.; Schwartzkopf M.; Hinz A.; Polonskyi O.; Strunskus T.; Faupel F.; Roth S. V.; Müller-Buschbaum P. Following in Situ the Deposition of Gold Electrodes on Low Band Gap Polymer Films. ACS Appl. Mater. Interfaces 2020, 12 (1), 1132–1141. 10.1021/acsami.9b17590. 31829550

[ref29] Kas R.; Yang K.; Bohra D.; Kortlever R.; Burdyny T.; Smith W. A. Electrochemical Co2 Reduction on Nanostructured Metal Electrodes: Fact or Defect?. Chem. Sci. 2020, 11 (7), 1738–1749. 10.1039/C9SC05375A. 34123269 PMC8150108

[ref30] Marcandalli G.; Goyal A.; Koper M. T. M. Electrolyte Effects on the Faradaic Efficiency of CO2Reduction to CO on a Gold Electrode. ACS Catal. 2021, 11 (9), 4936–4945. 10.1021/acscatal.1c00272. 34055454 PMC8154322

[ref31] Bandorf R.; Waschke S.; Carreri F. C.; Vergöhl M.; Grundmeier G.; Bräuer G. Direct Metallization of PMMA with Aluminum Films Using HIPIMS. Surf. Coat. Technol. 2016, 290, 77–81. 10.1016/j.surfcoat.2015.10.070.

[ref32] Bandorf R.; Waschke S.; Vergöhl M.; Grundmeier G.; Bräuer G. Direct Metallization of Plastics by High Power Impulse Magnetron Sputtering. Vak. Forsch. Prax. 2015, 27 (4), 18–23. 10.1002/vipr.201500587.

[ref33] Bulut Y.; Sochor B.; Harder C.; Reck K.; Drewes J.; Xu Z.; Jiang X.; Meinhardt A.; Jeromin A.; Kohantorabi M.; Noei H.; Keller T. F.; Strunskus T.; Faupel F.; Müller-Buschbaum P.; Roth S. V. Diblock Copolymer Pattern Protection by Silver Cluster Reinforcement. Nanoscale 2023, 15 (38), 15768–15774. 10.1039/D3NR03215A. 37740389

[ref34] Christou C.; Barber Z. H. Ionization of Sputtered Material in a Planar Magnetron Discharge. J. Vac. Sci. Technol. A 2000, 18 (6), 2897–2907. 10.1116/1.1312370.

[ref35] Lundin D.; Larsson P.; Wallin E.; Lattemann M.; Brenning N.; Helmersson U. Cross-Field Ion Transport during High Power Impulse Magnetron Sputtering. Plasma Sources Sci. Technol 2008, 17 (3), 035021 10.1088/0963-0252/17/3/035021.

[ref36] Lü B.; Münger E. P.; Sarakinos K. Coalescence-Controlled and Coalescence-Free Growth Regimes during Deposition of Pulsed Metal Vapor Fluxes on Insulating Surfaces. J. Appl. Phys. 2015, 117 (13), 134304 10.1063/1.4916983.

[ref37] Magnfält D.; Elofsson V.; Abadias G.; Helmersson U.; Sarakinos K. Time-Domain and Energetic Bombardment Effects on the Nucleation and Coalescence of Thin Metal Films on Amorphous Substrates. J. Phys. D: Appl. Phys. 2013, 46 (21), 215303 10.1088/0022-3727/46/21/215303.

[ref38] Schwartzkopf M.; Hinz A.; Polonskyi O.; Strunskus T.; Löhrer F. C.; Körstgens V.; Müller-Buschbaum P.; Faupel F.; Roth S. V. Role of Sputter Deposition Rate in Tailoring Nanogranular Gold Structures on Polymer Surfaces. ACS Appl. Mater. Interfaces 2017, 9 (6), 5629–5637. 10.1021/acsami.6b15172. 28106380

[ref39] Schwartzkopf M.; Santoro G.; Brett C. J.; Rothkirch A.; Polonskyi O.; Hinz A.; Metwalli E.; Yao Y.; Strunskus T.; Faupel F.; Müller-Buschbaum P.; Roth S. V. Real-Time Monitoring of Morphology and Optical Properties during Sputter Deposition for Tailoring Metal–Polymer Interfaces. ACS Appl. Mater. Interfaces 2015, 7 (24), 13547–13556. 10.1021/acsami.5b02901. 26030314

[ref40] Amarandei G.; O’Dwyer C.; Arshak A.; Corcoran D. The Stability of Thin Polymer Films as Controlled by Changes in Uniformly Sputtered Gold. Soft Matter 2013, 9 (9), 2695–2702. 10.1039/c3sm27130g.

[ref41] Schwartzkopf M.; Buffet A.; Körstgens V.; Metwalli E.; Schlage K.; Benecke G.; Perlich J.; Rawolle M.; Rothkirch A.; Heidmann B.; Herzog G.; Müller-Buschbaum P.; Röhlsberger R.; Gehrke R.; Stribeck N.; Roth S. V. From Atoms to Layers: In Situ Gold Cluster Growth Kinetics during Sputter Deposition. Nanoscale 2013, 5 (11), 5053–5062. 10.1039/c3nr34216f. 23640164

[ref42] Stierle A.; Keller T. F.; Noei H.; Vonk V.; Roehlsberger R. DESY NanoLab. J. Large-Scale Res. Facil. 2016, 2 (A76), A76 10.17815/jlsrf-2-140.

[ref43] Schneider C. A.; Rasband W. S.; Eliceiri K. W. NIH Image to ImageJ: 25 Years of Image Analysis. Nat. Methods 2012, 9 (7), 671–675. 10.1038/nmeth.2089. 22930834 PMC5554542

[ref44] Benecke G.; Wagermaier W.; Li C.; Schwartzkopf M.; Flucke G.; Hoerth R.; Zizak I.; Burghammer M.; Metwalli E.; Müller-Buschbaum P.; Trebbin M.; Förster S.; Paris O.; Roth S. V.; Fratzl P. A Customizable Software for Fast Reduction and Analysis of Large X-Ray Scattering Data Sets: Applications of the New DPDAK Package to Small-Angle X-Ray Scattering and Grazing-Incidence Small-Angle X-Ray Scattering. J. Appl. Crystallogr. 2014, 47 (5), 1797–1803. 10.1107/S1600576714019773. 25294982 PMC4180741

[ref45] Nelson A. R. J.; Prescott S. W. Refnx: Neutron and X-Ray Reflectometry Analysis in Python. J. Appl. Crystallogr. 2019, 52, 193–200. 10.1107/S1600576718017296. 30800030 PMC6362611

[ref46] Kaune G.; Ruderer M. A.; Metwalli E.; Wang W.; Couet S.; Schlage K.; Röhlsberger R.; Roth S. V.; Müller-Buschbaum P. Situ GISAXS Study of Gold Film Growth on Conducting Polymer Films. ACS Appl. Mater. Interfaces 2009, 1 (2), 353–360. 10.1021/am8000727. 20353223

[ref47] Venables J. A.; Spiller G. D. T.; Hanbucken M. Nucleation and Growth of Thin Films. Rep. Prog. Phys. 1984, 47 (4), 399–459. 10.1088/0034-4885/47/4/002.

[ref48] Ehrlich G. Direct Observations of the Surface Diffusion of Atoms and Clusters. Surf. Sci 1991, 246 (1–3), 1–12. 10.1016/0039-6028(91)90385-6.

[ref49] Faupel F.; Zaporojtchenko V.; Strunskus T.; Elbahri M. Metal-Polymer Nanocomposites for Functional Applications. Adv. Eng. Mater. 2010, 12 (12), 1177–1190. 10.1002/adem.201000231.

[ref50] Reck K. A.; Bulut Y.; Xu Z.; Liang S.; Strunskus T.; Sochor B.; Gerdes H.; Bandorf R.; Müller-Buschbaum P.; Roth S. V.; Vahl A.; Faupel F. Early-Stage Silver Growth during Sputter Deposition on SiO2 and Polystyrene – Comparison of Biased DC Magnetron Sputtering, High-Power Impulse Magnetron Sputtering (HiPIMS) and Bipolar HiPIMS. Appl. Surf. Sci. 2024, 666 (May), 160392 10.1016/j.apsusc.2024.160392.

[ref51] Gensch M.; Schwartzkopf M.; Brett C. J.; Schaper S. J.; Kreuzer L. P.; Li N.; Chen W.; Liang S.; Drewes J.; Polonskyi O.; Strunskus T.; Faupel F.; Müller-Buschbaum P.; Roth S. V. Selective Silver Nanocluster Metallization on Conjugated Diblock Copolymer Templates for Sensing and Photovoltaic Applications. ACS Appl. Nano Mater. 2021, 4 (4), 4245–4255. 10.1021/acsanm.1c00829.

[ref52] Schaper S. J.; Löhrer F. C.; Xia S.; Geiger C.; Schwartzkopf M.; Pandit P.; Rubeck J.; Fricke B.; Frenzke S.; Hinz A. M.; Carstens N.; Polonskyi O.; Strunskus T.; Faupel F.; Roth S. V.; Müller-Buschbaum P. Revealing the Growth of Copper on Polystyrene- Block -Poly(Ethylene Oxide) Diblock Copolymer Thin Films with in Situ GISAXS. Nanoscale 2021, 13 (23), 10555–10565. 10.1039/D1NR01480C. 34100512

[ref53] Williamson G. K.; Hall W. H. X-Ray Line Broadening from Filed Aluminium and Wolfram. Acta Metall. 1953, 1 (1), 22–31. 10.1016/0001-6160(53)90006-6.

[ref54] Zou Y.; Eichhorn J.; Zhang J.; Apfelbeck F. A. C.; Yin S.; Wolz L.; Chen C. C.; Sharp I. D.; Müller-Buschbaum P. Microstrain and Crystal Orientation Variation within Naked Triple-Cation Mixed Halide Perovskites under Heat, UV, and Visible Light Exposure. ACS Energy Lett. 2024, 9 (2), 388–399. 10.1021/acsenergylett.3c02617. 38356935 PMC10863397

[ref55] Milligan W. O.; Morriss R. H. Morphology of Colloidal Gold--A Comparative Study. J. Am. Chem. Soc. 1964, 86 (17), 3461–3467. 10.1021/ja01071a012.

[ref56] Davey W. P. Precision Measurements of the Lattice Constants of Twelve Common Metals. Phys. Rev. 1925, 25 (6), 753–761. 10.1103/PhysRev.25.753.

