# Supplementary material for: Investigating Gold Deposition with High-Power Impulse Magnetron Sputtering and Direct-Current Magnetron Sputtering on Polystyrene, Poly-4-vinylpyridine, and Polystyrene Sulfonic Acid
Source: Langmuir. 2024 Oct 15;40(43):22591–601. doi: 10.1021/acs.langmuir.4c02344 (PMC11526365; doi:10.1021/acs.langmuir.4c02344)
Supplement: Supplementary file 1 — la4c02344_si_001.pdf [file la4c02344_si_001.pdf]

Supporting Information

Investigating Gold Deposition with High Power Impulse Magnetron Sputtering and Direct Current Magnetron Sputtering on Polystyrene, Poly-4-vinylpyridine and Polystyrene Sulfonic Acid

Yusuf Bulut[†,‡], *Benedikt Sochor*[†], *Kristian A. Reck*[#], *Bernhard Schummer*[§], *Alexander Meinhardt*[$,∥], *Jonas Drewes*[#], *Suzhe Liang*[‡], *Tianfu Guan*[‡], *Arno Jeromin*[$],*Andreas Stierle*[$,∥], *Thomas F. Keller*[$,∥], *Thomas Strunskus*[#], *Franz Faupel*[#], *Peter Müller-Buschbaum*[‡,∇], *Stephan V. Roth*[*,†,⊥]

[†]Deutsches Elektronen-Synchrotron DESY, Notkestr. 85, 22607 Hamburg, Germany

[‡]Technical University of Munich, TUM School of Natural Sciences, Department of Physics, Chair for Functional Materials, James-Franck-Str. 1, 85748 Garching, Germany

[§]Fraunhofer Institute for Integrated Circuits IIS, Development Center for X-ray Technology EZRT, Flugplatzstr. 75, 90768 Fürth, Germany

[$]Centre for X-ray and Nano Science CXNS, Deutsches Elektronen-Synchrotron DESY, Notkestr. 85, 22607 Hamburg, Germany

[∥]Department of Physics, University of Hamburg, Notkestr. 9-11, 22607 Hamburg, Germany

[#] Chair for Multicomponent Materials, Department for Materials Science, Faculty of Engineering, Kiel University, Kaiserstr. 2, 24143 Kiel, Germany

[∇]Heinz Maier-Leibnitz Zentrum (MLZ), Technical University of Munich, Lichtenbergstraße 1, 85748 Garching, Germany

[⊥]KTH Royal Institute of Technology, Teknikringen 56-58, 100 44 Stockholm, Sweden

*Email address: stephan.roth@desy.de;svroth@kth.se

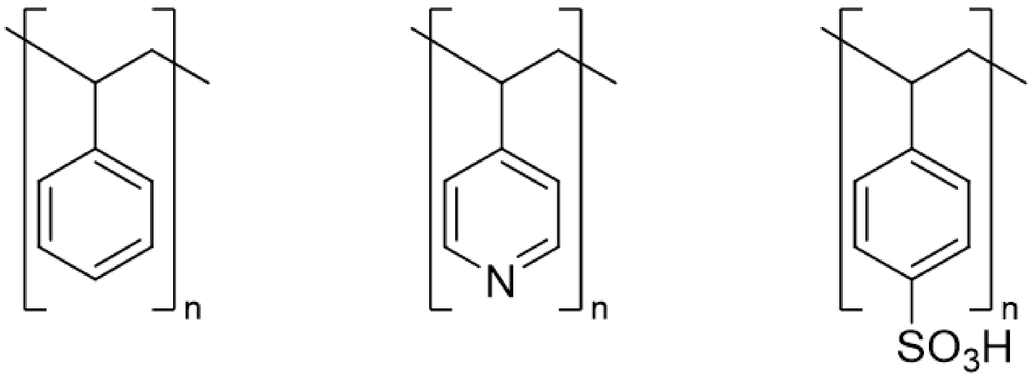

Polystyrene $[C_8H_8]_n$

Poly-4-vinylpyridine $[C_8H_7N]_n$

Polystyrenesulfonicacid $[C_8H_8SO_3]_n$

Figure S1: Displayed is the skeletal structure of polystyrene (PS), poly-4-vinylpyridine (P4VP) and polystyrene sulfonic acid (PSS).

For the XRR analysis Motofit was used utilizing the standard slab model using the Parratt formalism.[1] Silicon was used as a substrate. On top of the silicon is a native SiO_x layer. On top of the native SiO_x layer is the corresponding polymer layer.

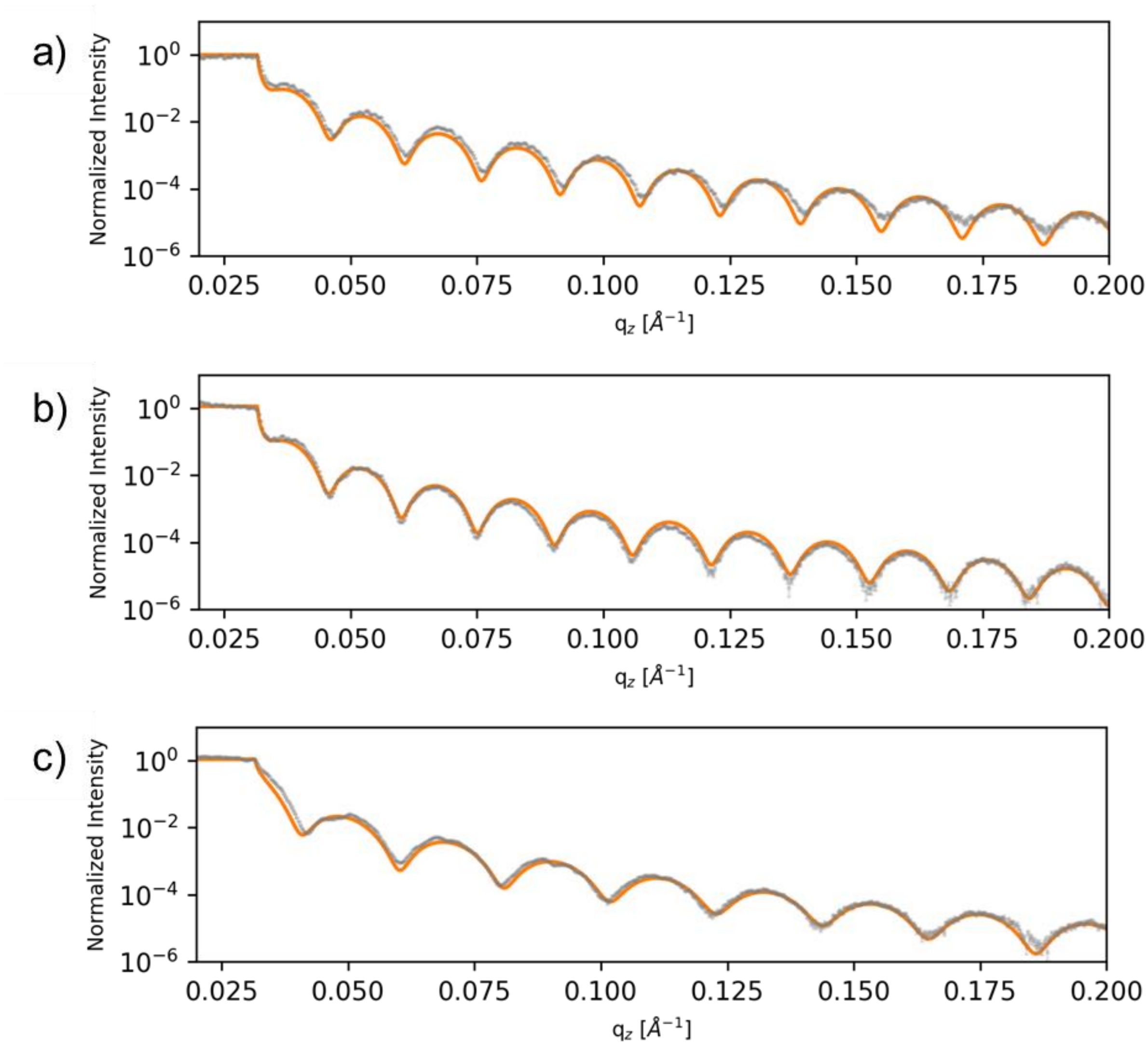

Figure S2: XRR measurements (grey symbols) with corresponding fits (orange line) of (a) PS, (b) P4VP and (c) PSS.

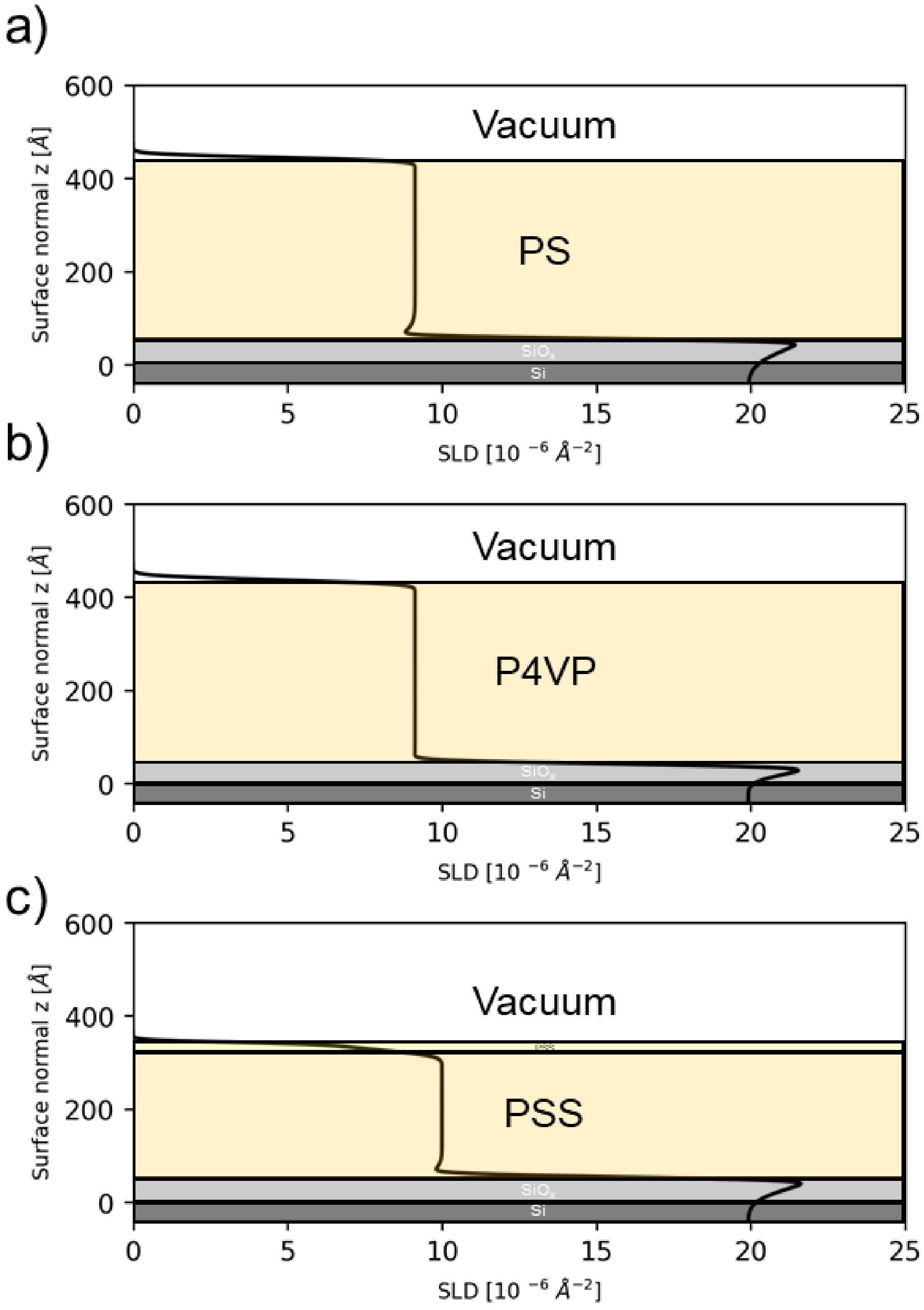

Figure S3: SLD profile of (a) PS, (b) (P4VP) and (c) PSS.

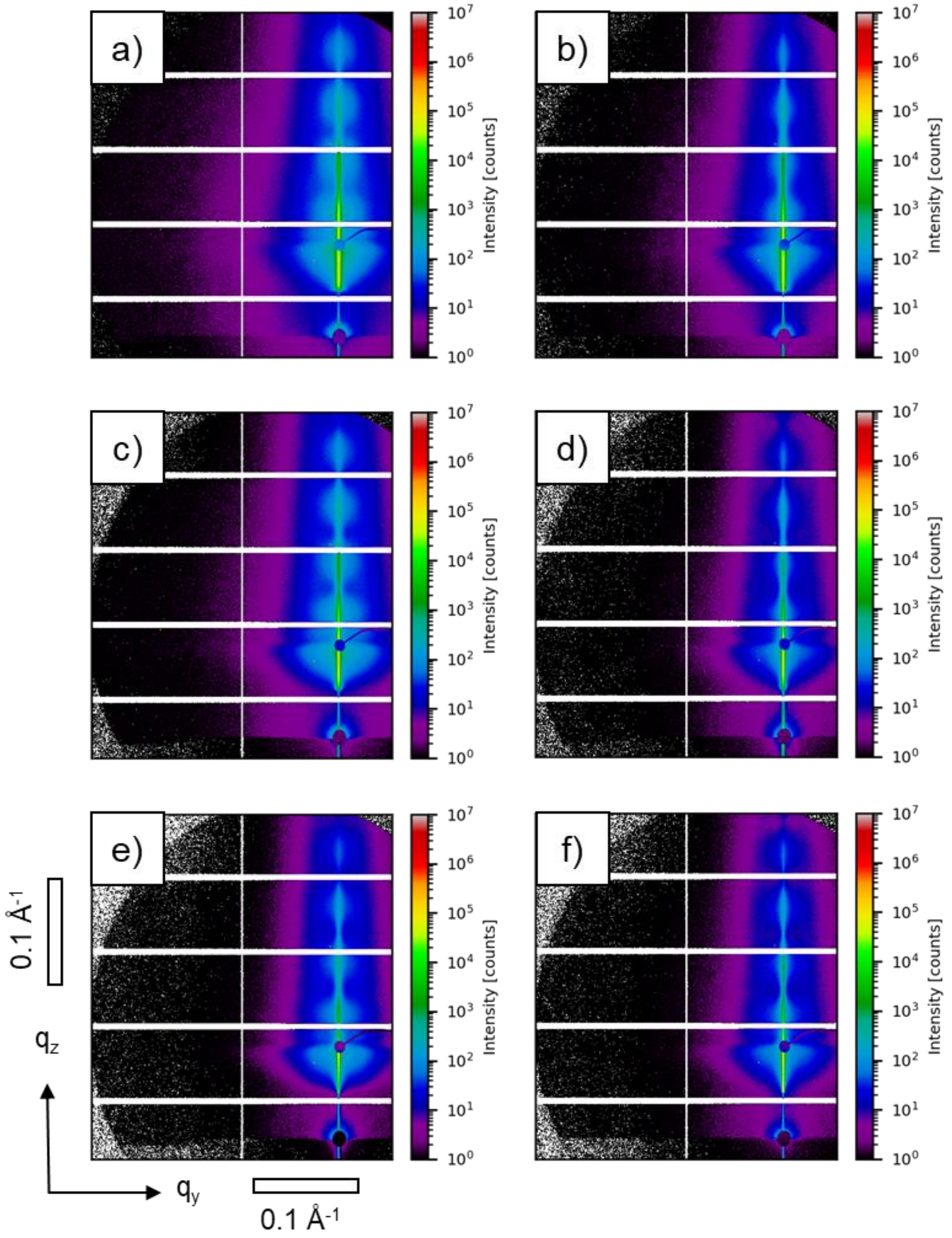

Figure S4: Raw GISXAS detector images of the in-situ sequences of (a) Au:PS$_{dcMS}$, (b) Au:PS$_{HiPIMS}$, (c) Au:P4VP$_{dcMS}$, (d) Au:P4VP$_{HiPIMS}$, (e) Au:PSS$_{dcMS}$ and (f) Au:PSS$_{HiPIMS}$. From these detector images the final thickness was determined extracting the adjacent vertical peaks in the q_z direction and the relation $\delta=2\pi/\Delta q_z$.[3] This resulted in the final deposited thickness of

Au:PS$_{dcMS}$ = 11.7 ± 0.3 nm, Au:PS$_{HiPIMS}$ = 11.8 ± 0.3 nm, Au:P4VP$_{dcMS}$= 12.1 ± 0.3 nm, Au:P4VP$_{HiPIMS}$ = 12.3 ± 0.3 nm, Au:PSS$_{dcMS}$ = 11.7 ± 0.3 nm and Au:PSS$_{HiPIMS}$ = 12.0 ± 0.3 nm.

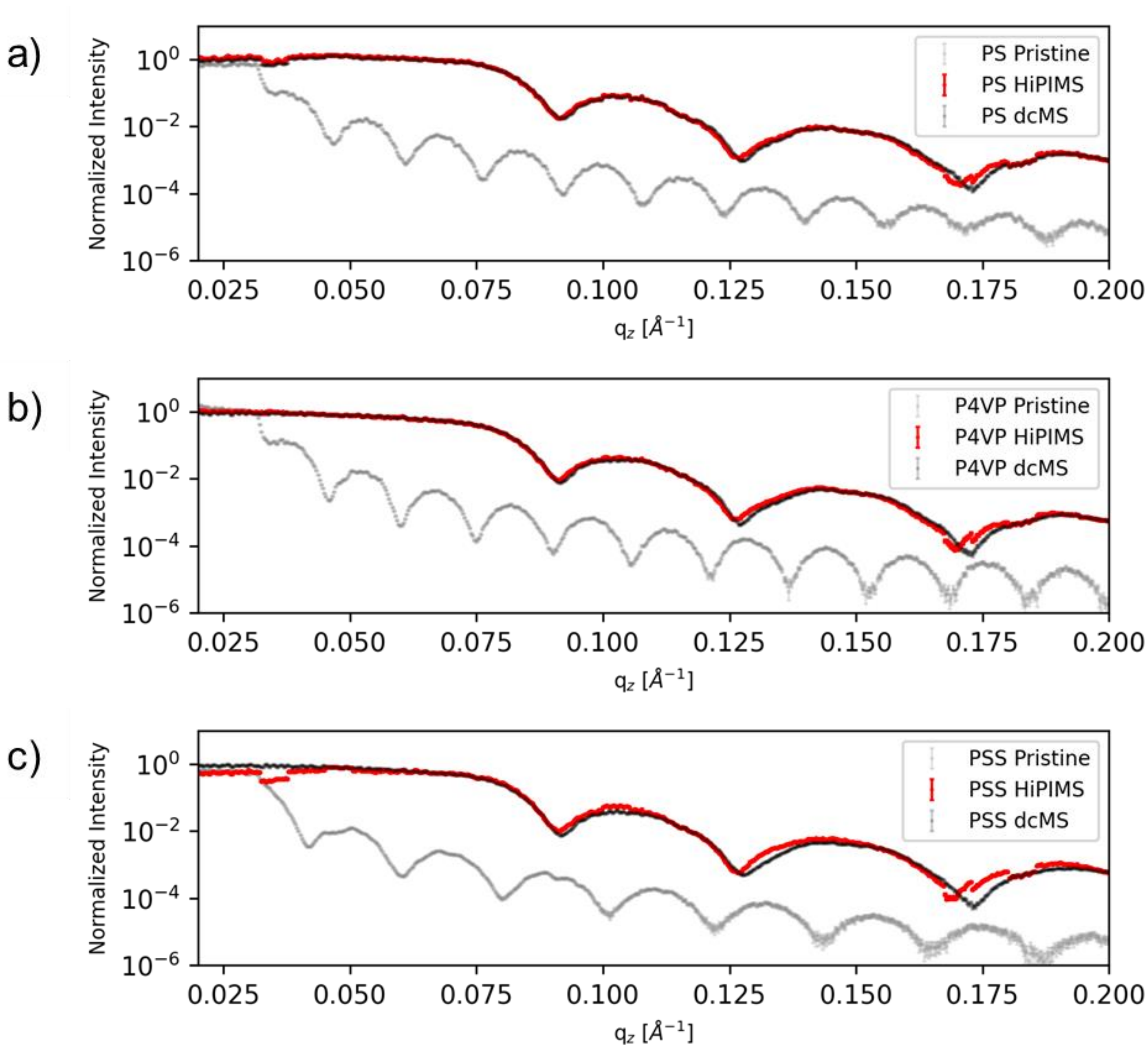

Figure S5: XRR measurements of (a) PS, (b) P4VP and (c) PSS under (red) HiPIMS and (darkgrey) dcMS conditions. The pristine polymer is shifted down along the Y-axis for improved visualization. The thickness of the granular Au layer is extracted of the minima with $2\pi/\Delta q_z$. The granular Au thickness are Au:PS$_{HiPIMS}$ = 12.3 ± 0.8 nm, Au:PS$_{dcMS}$ = 12.1 ± 0.7 nm, Au:P4VP$_{HiPIMS}$ = 12.3 ± 0.6 nm. Au:P4VP$_{dcMS}$= 12.4 ± 0.5 nm, Au:PSS$_{HiPIMS}$ = 12.4 ± 0.6 nm and Au:PSS$_{dcMS}$ =

12.1 ± 0.6 nm. The film thicknesses are large enough that silicon substrate effects can be neglected.[2]

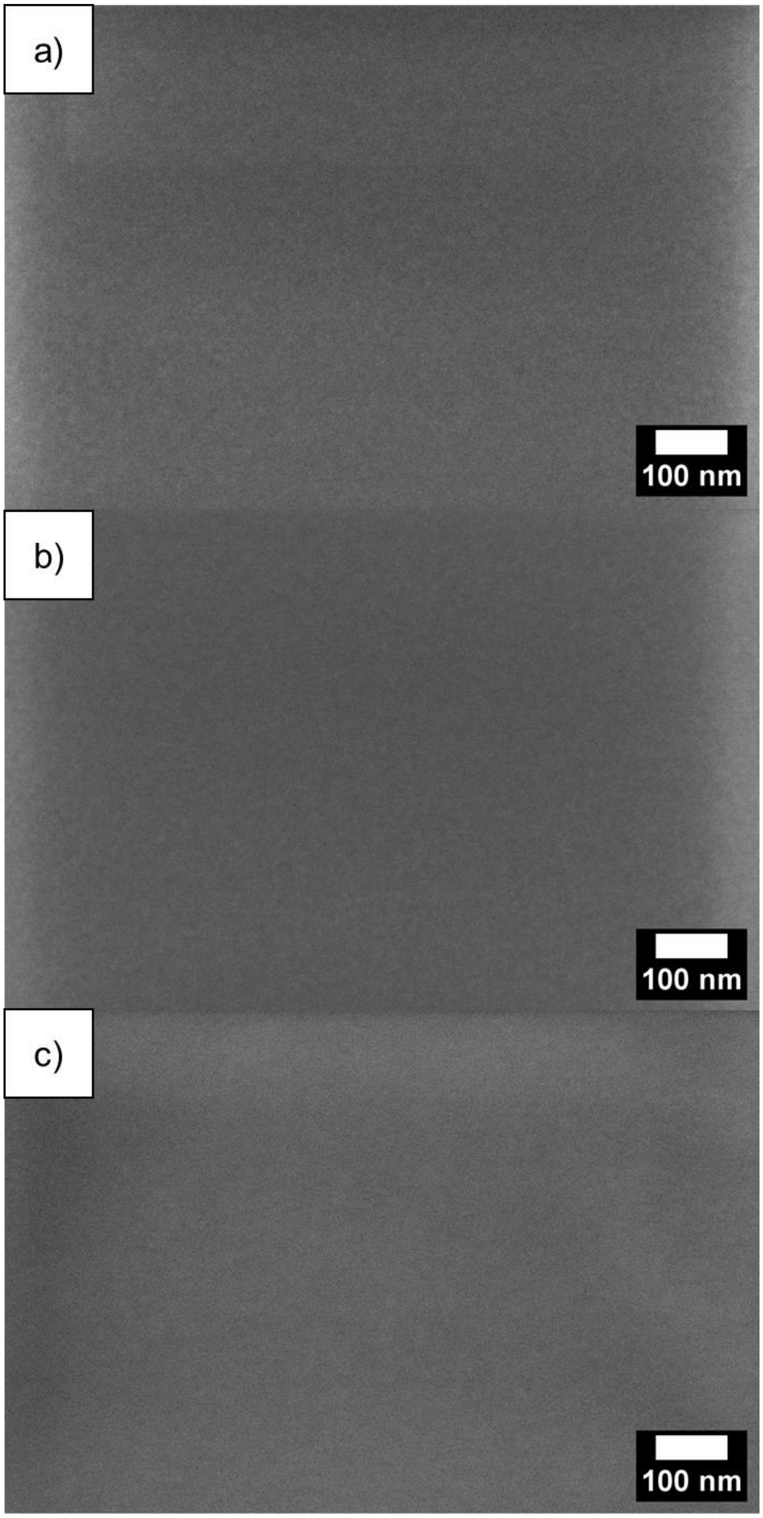

Figure S6: SEM measurements of pristine (a) PS, (b) P4VP and (c) PSS.

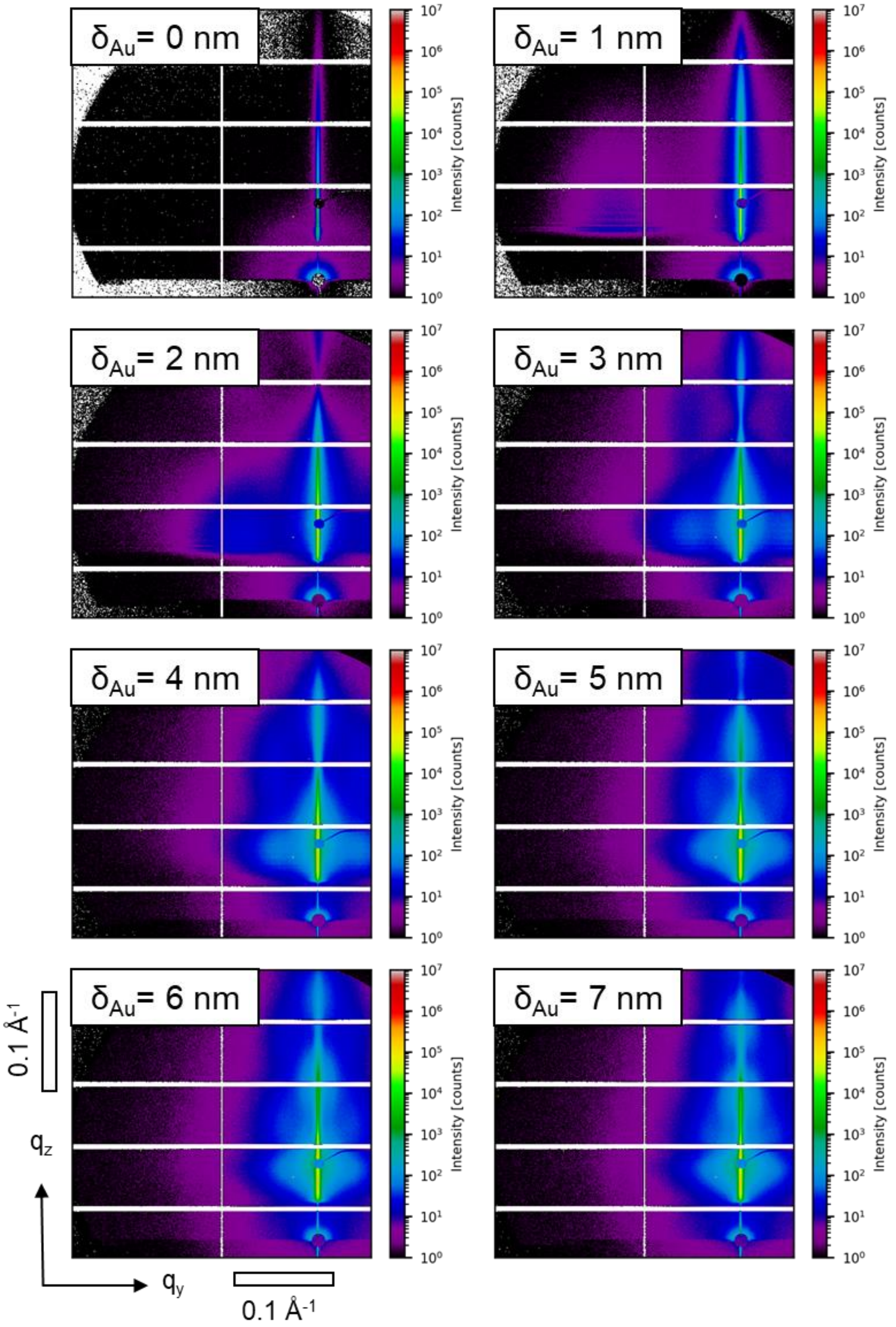

Figure S7: Raw GISAXS images of Au:PS$_{dcMS}$.

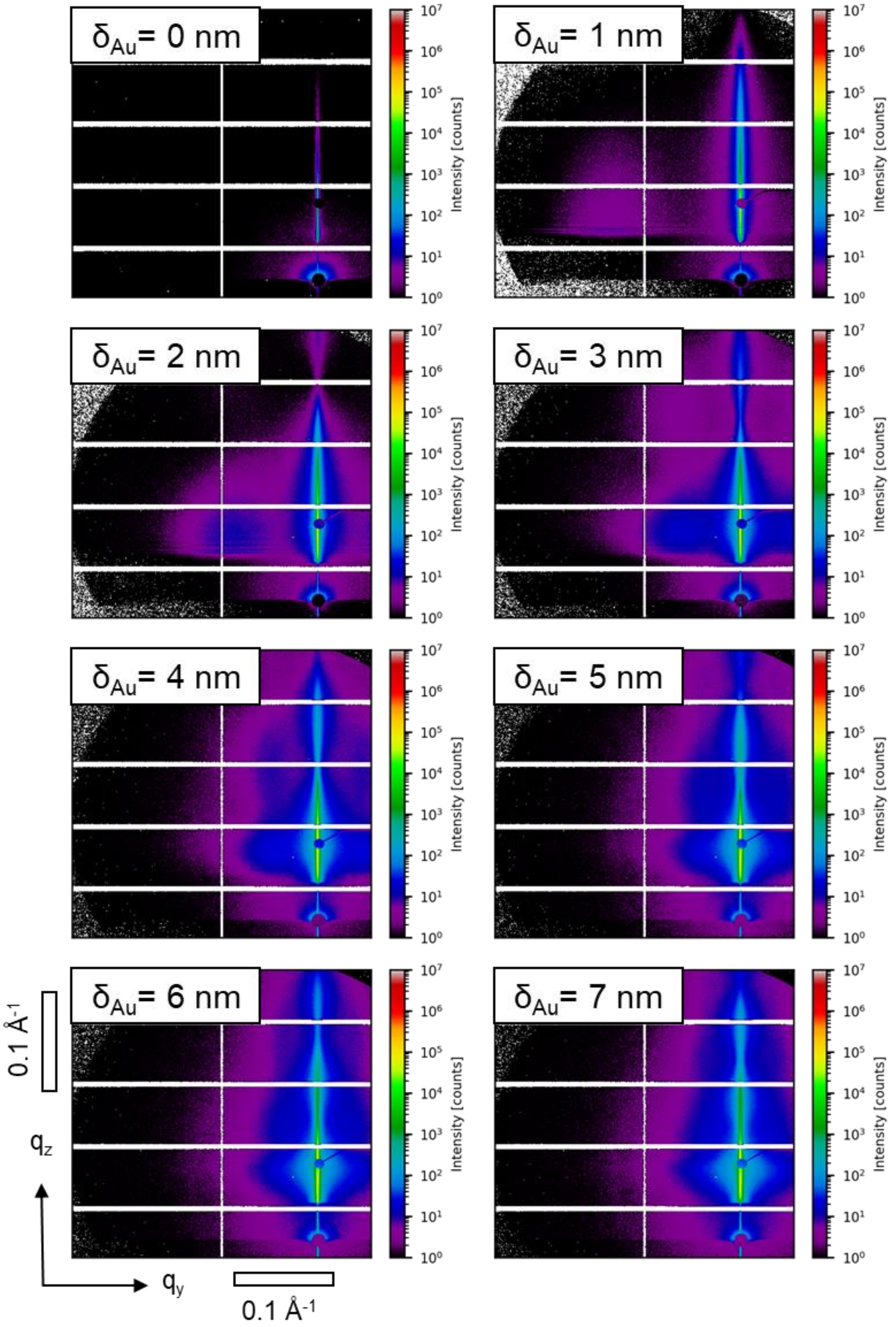

Figure S8: Raw GISAXS images of Au:PS_{HiPIMS}.

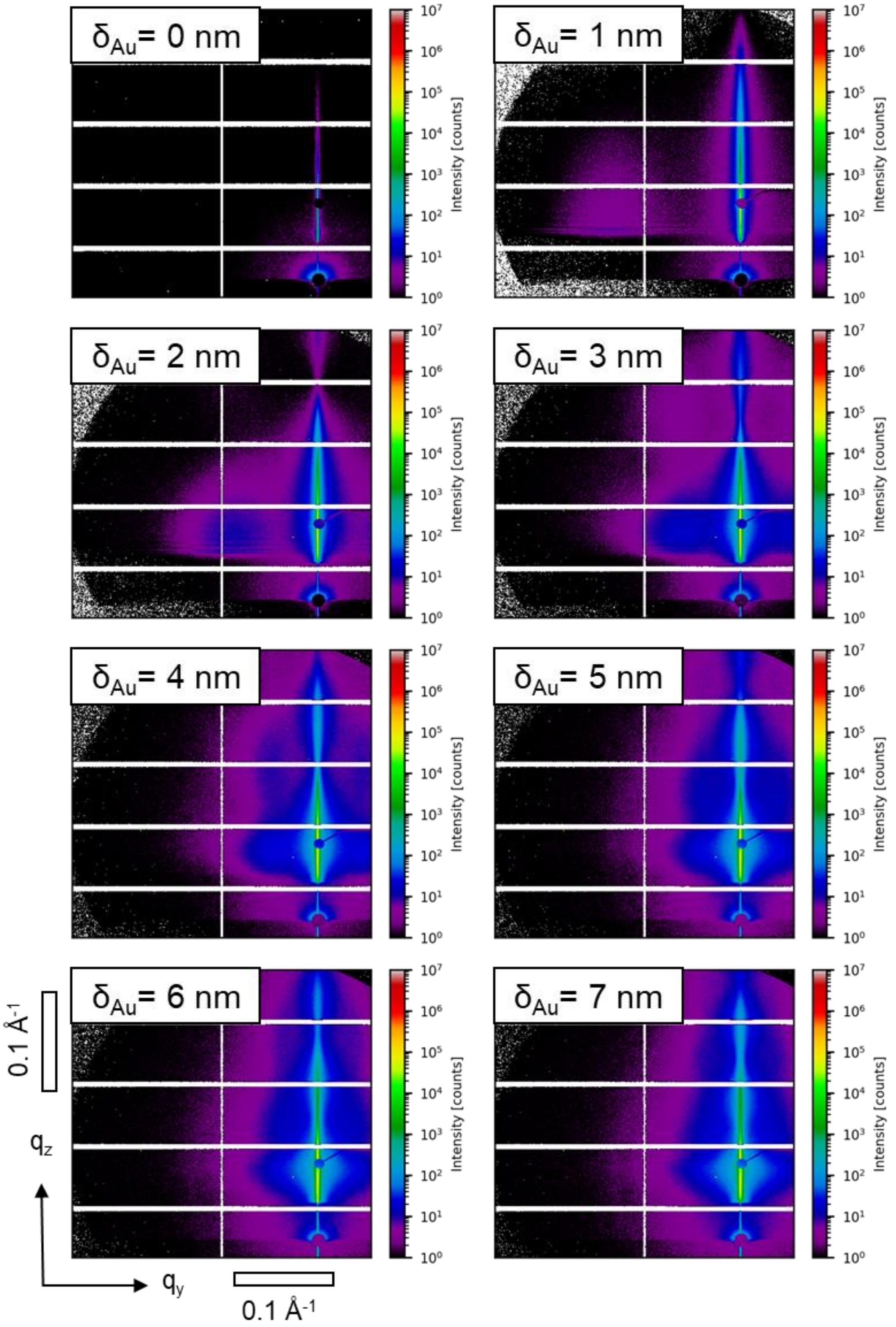

Figure S9: Raw GISAXS images of Au:P4VP$_{dcMS}$.

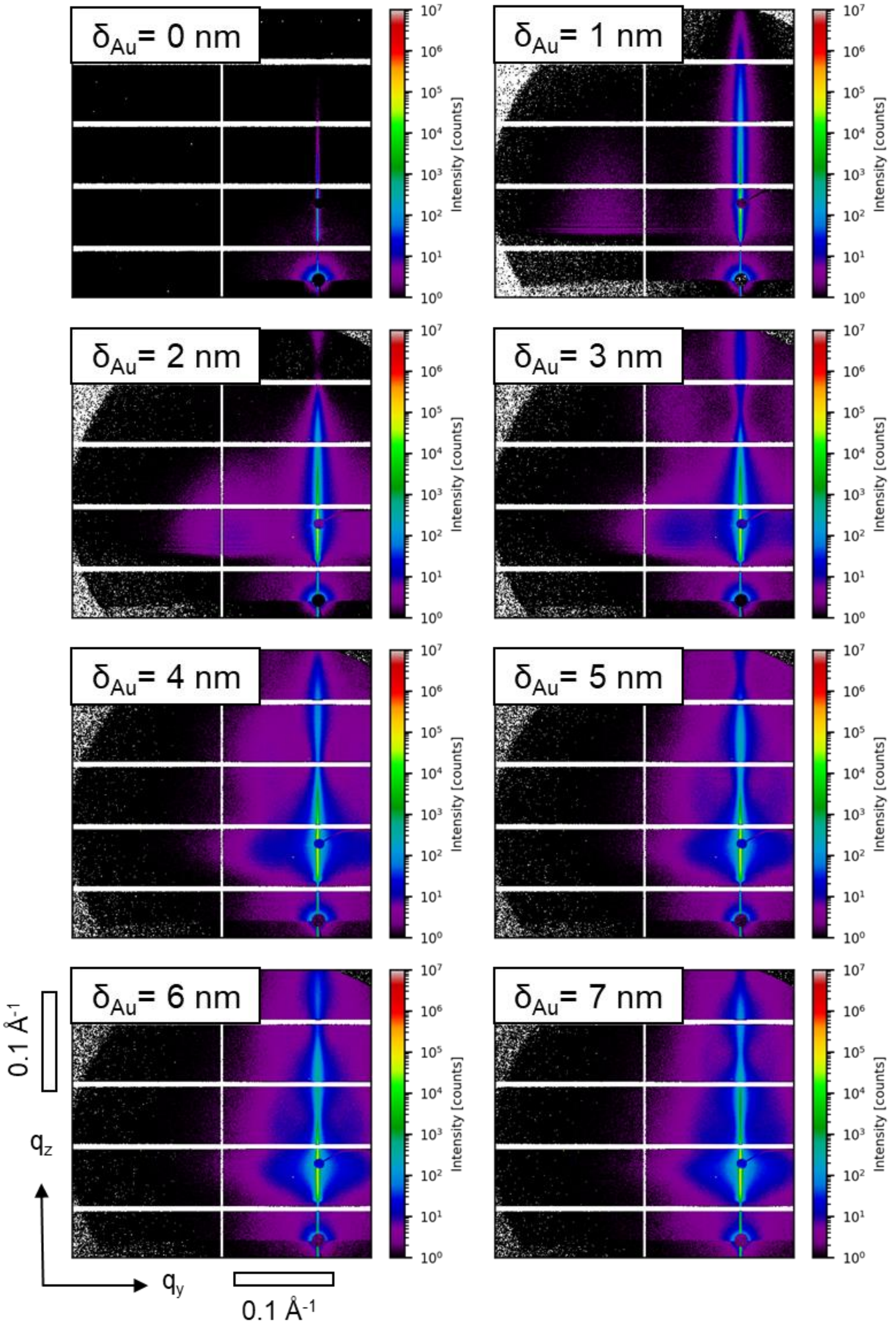

Figure S10: Raw GISAXS images of Au:P4VP$_{HiPIMS}$.

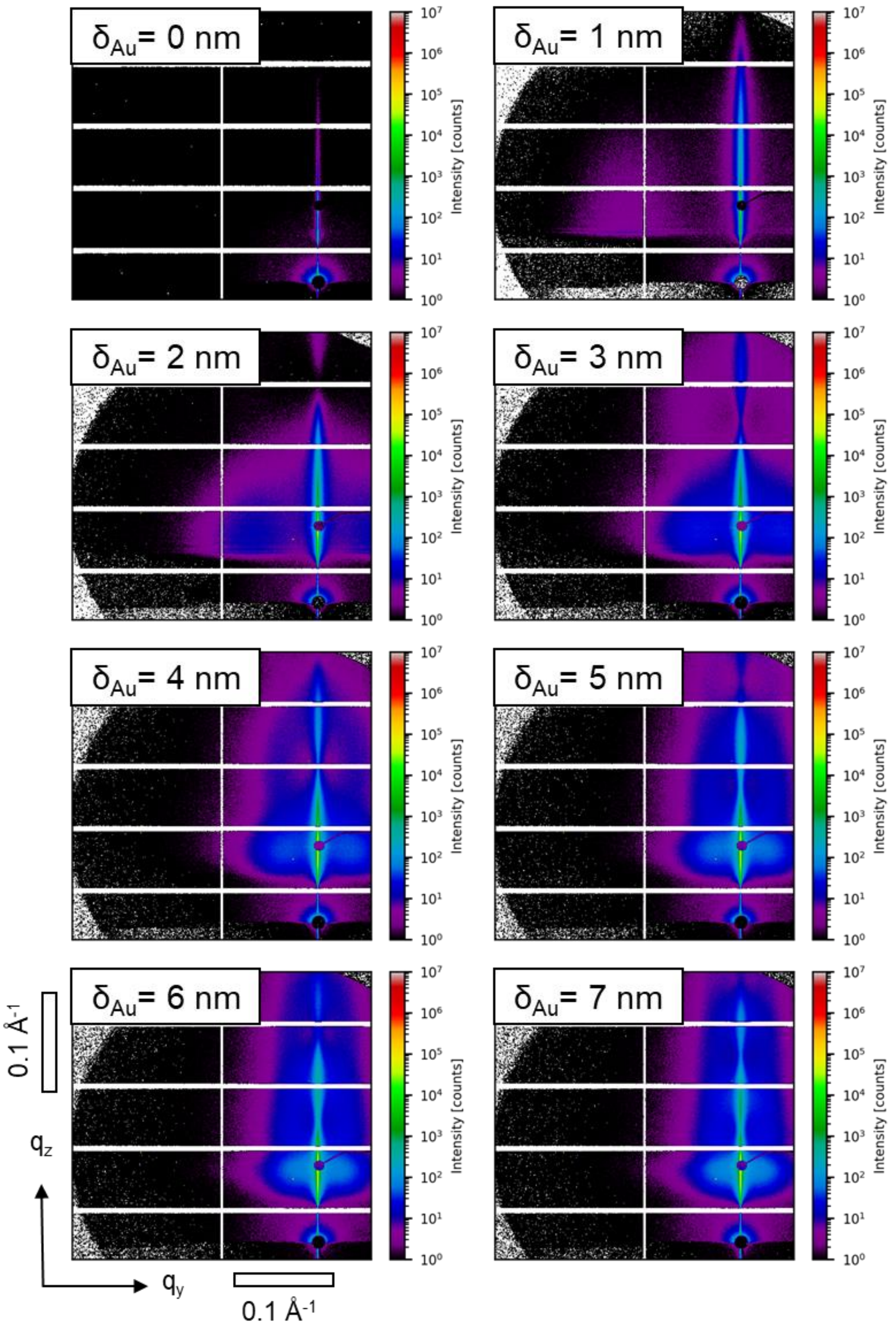

Figure S11: Raw GISAXS images of Au:PSS_{dcMS}.

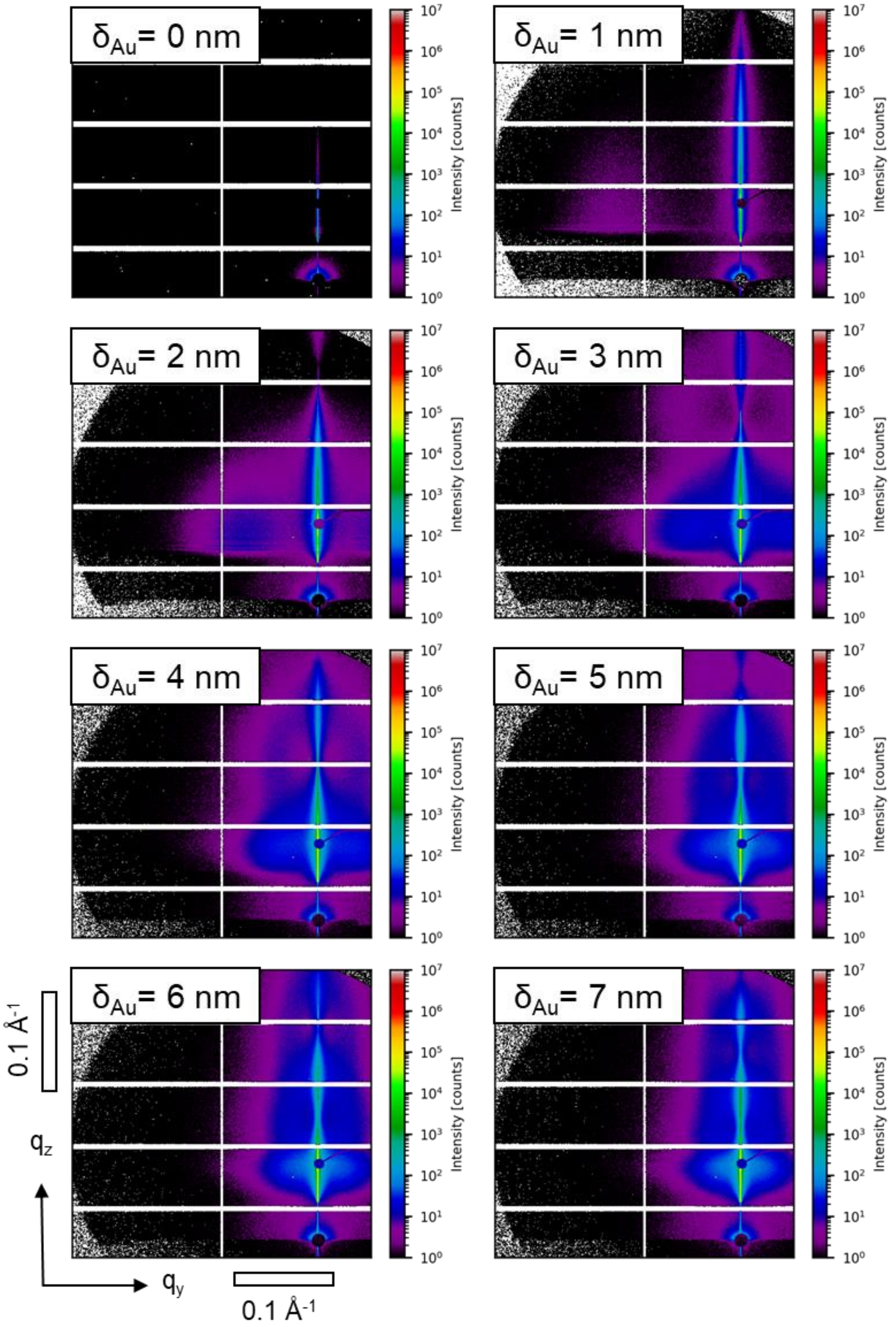

Figure S12: Raw GISAXS images of Au:$PSS_{HiPIMS.}$

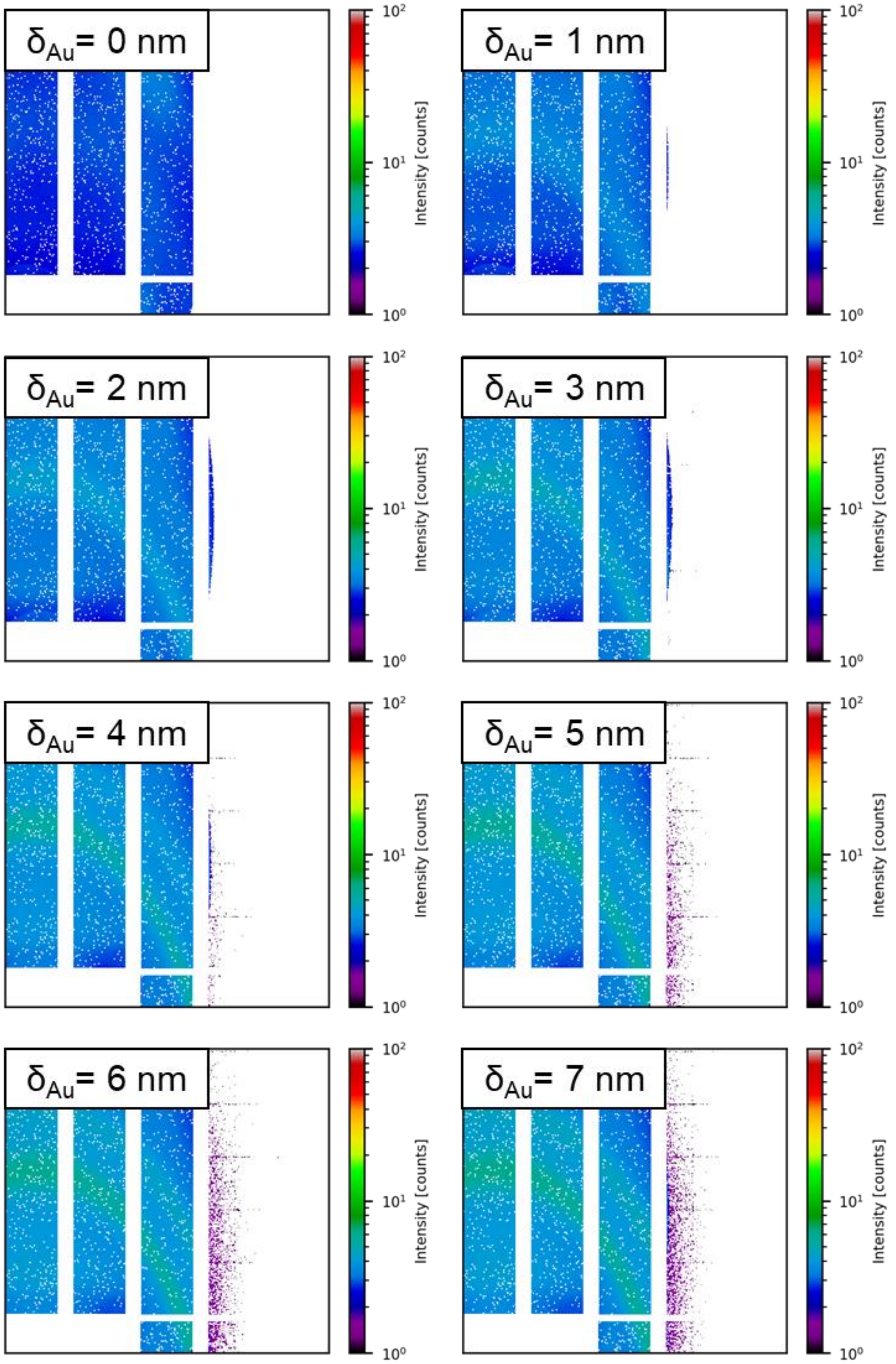

Figure S13: Raw GIWAXS images of Au:PS_{dcMS}.

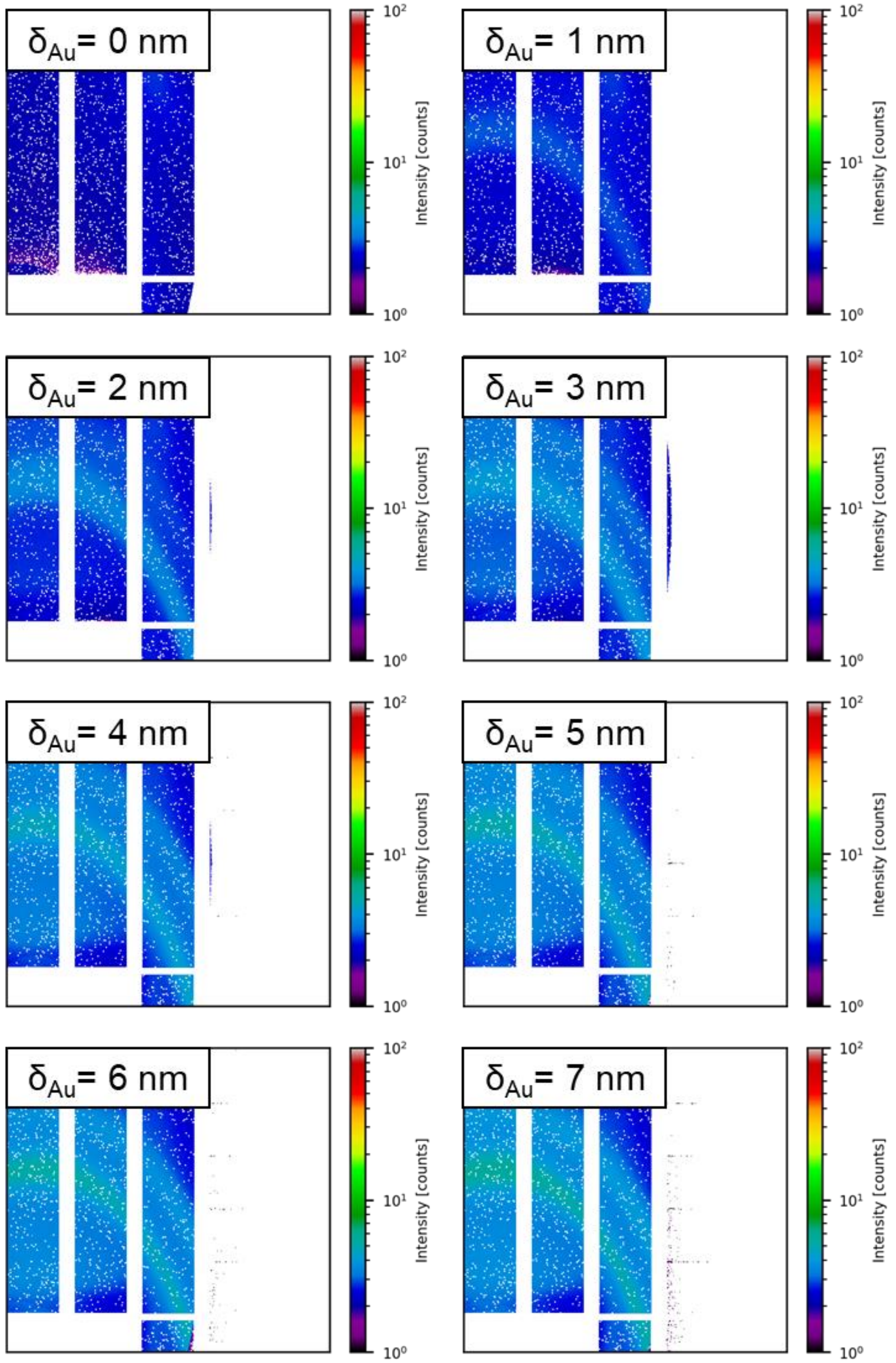

Figure S14: Raw GIWAXS images of Au:PS$_{HiPIMS}$.

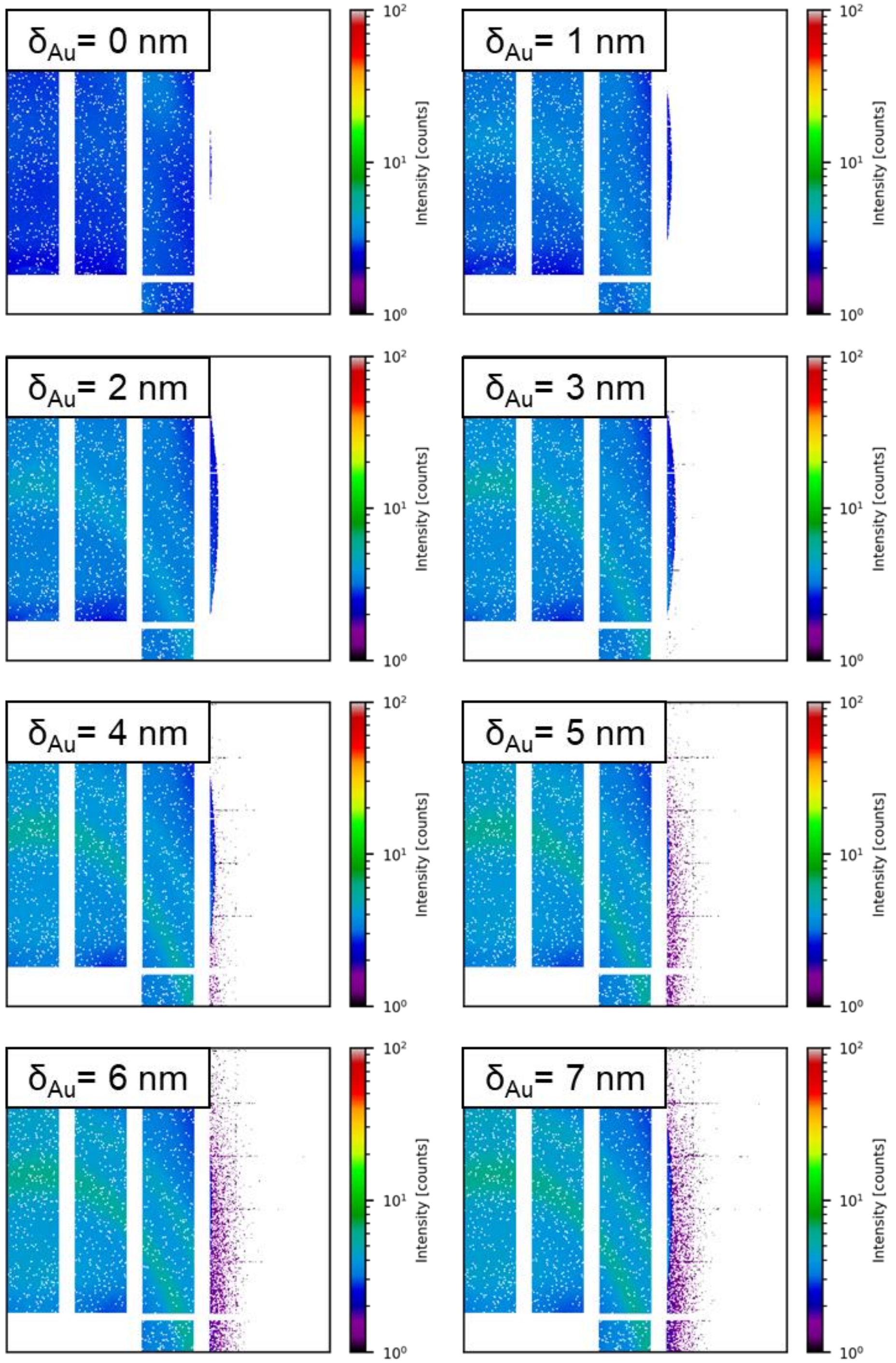

Figure S15: Raw GIWAXS images of Au:P4VP$_{dcMS}$.

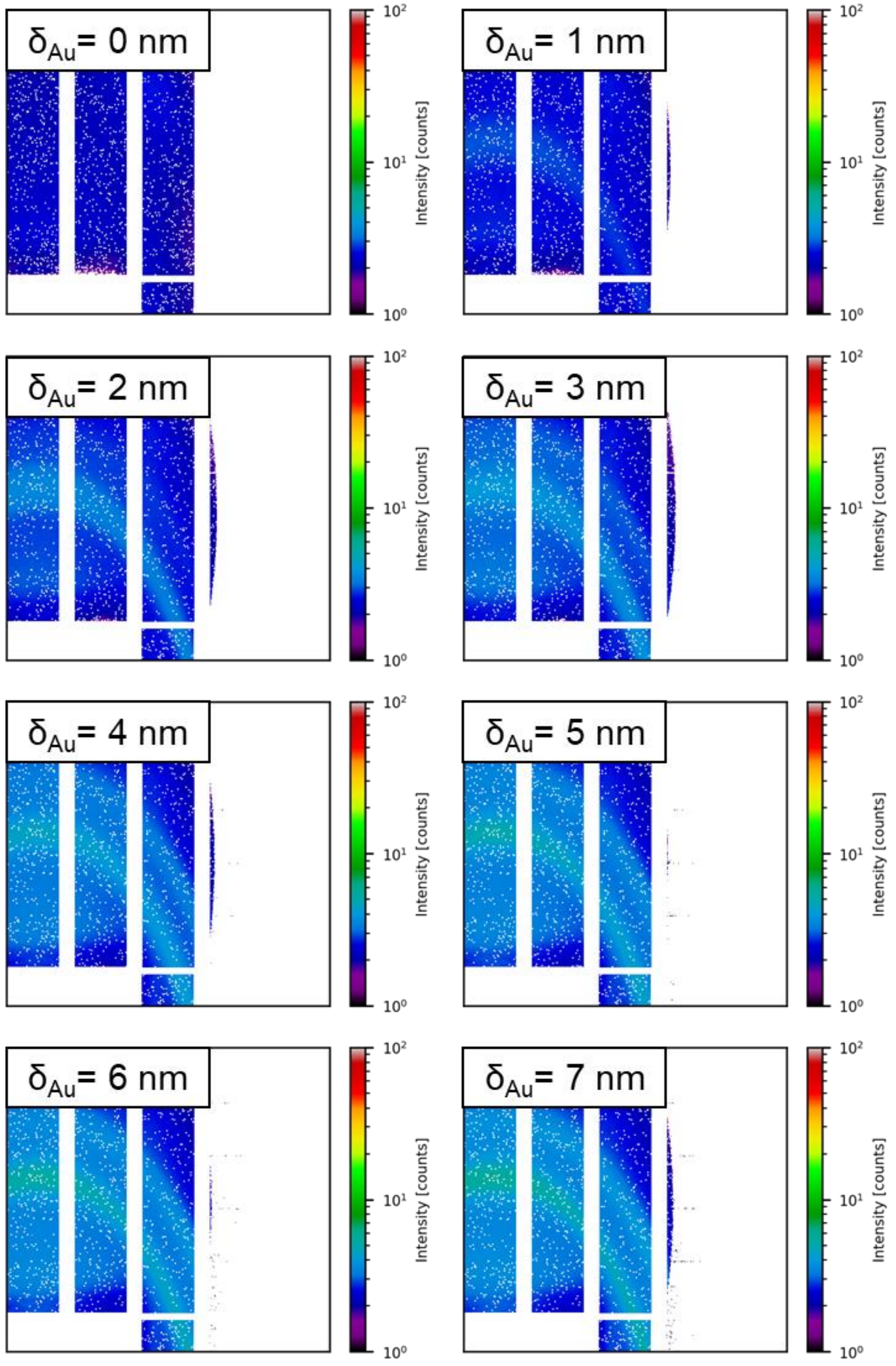

Figure S16: Raw GIWAXS images of Au:P4VP$_{HiPIMS}$.

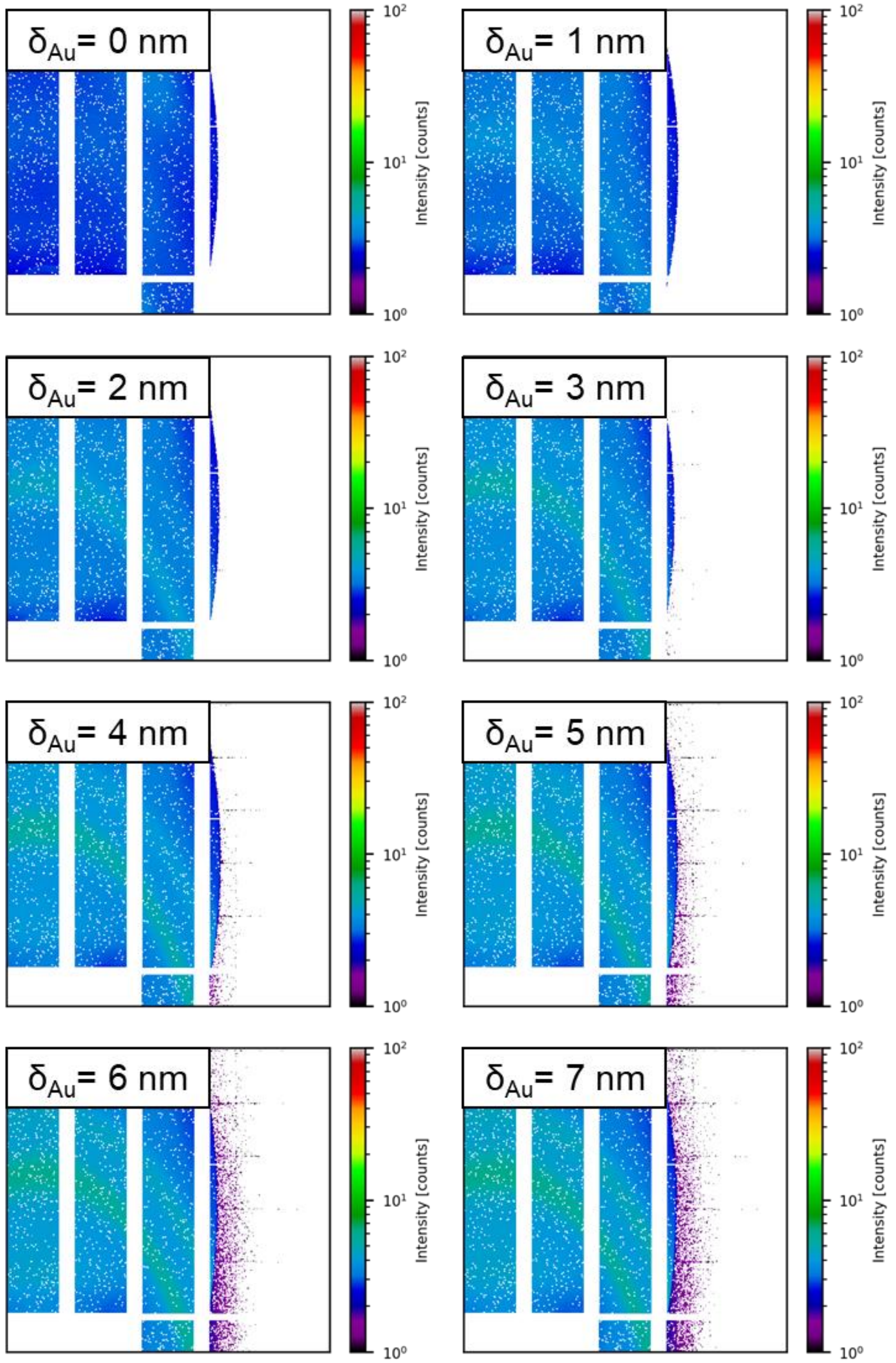

Figure S17: Raw GIWAXS images of Au:PSS_{dcMS}.

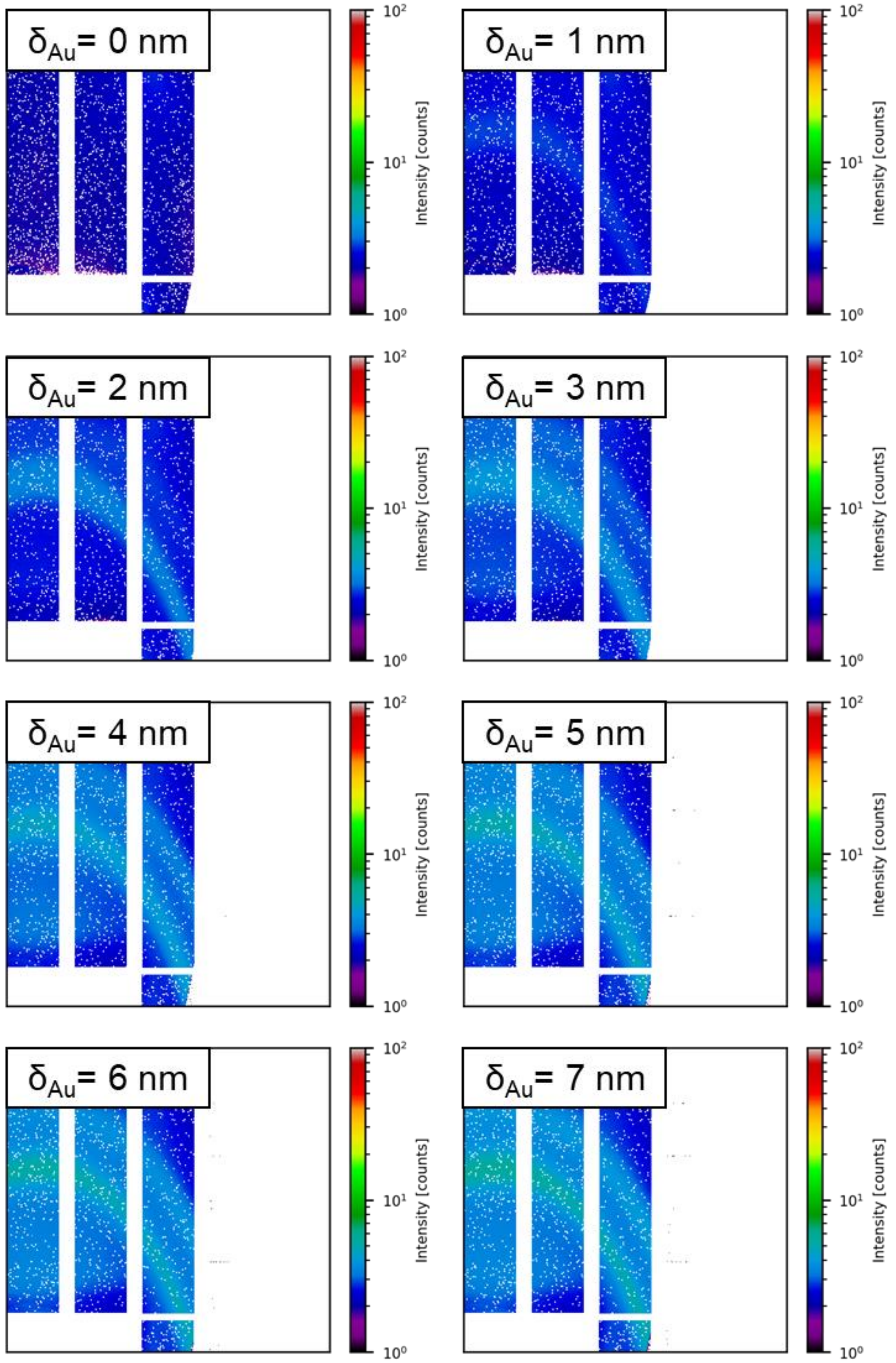

Figure S18: Raw GIWAXS images of Au:PSS_{HiPIMS}.

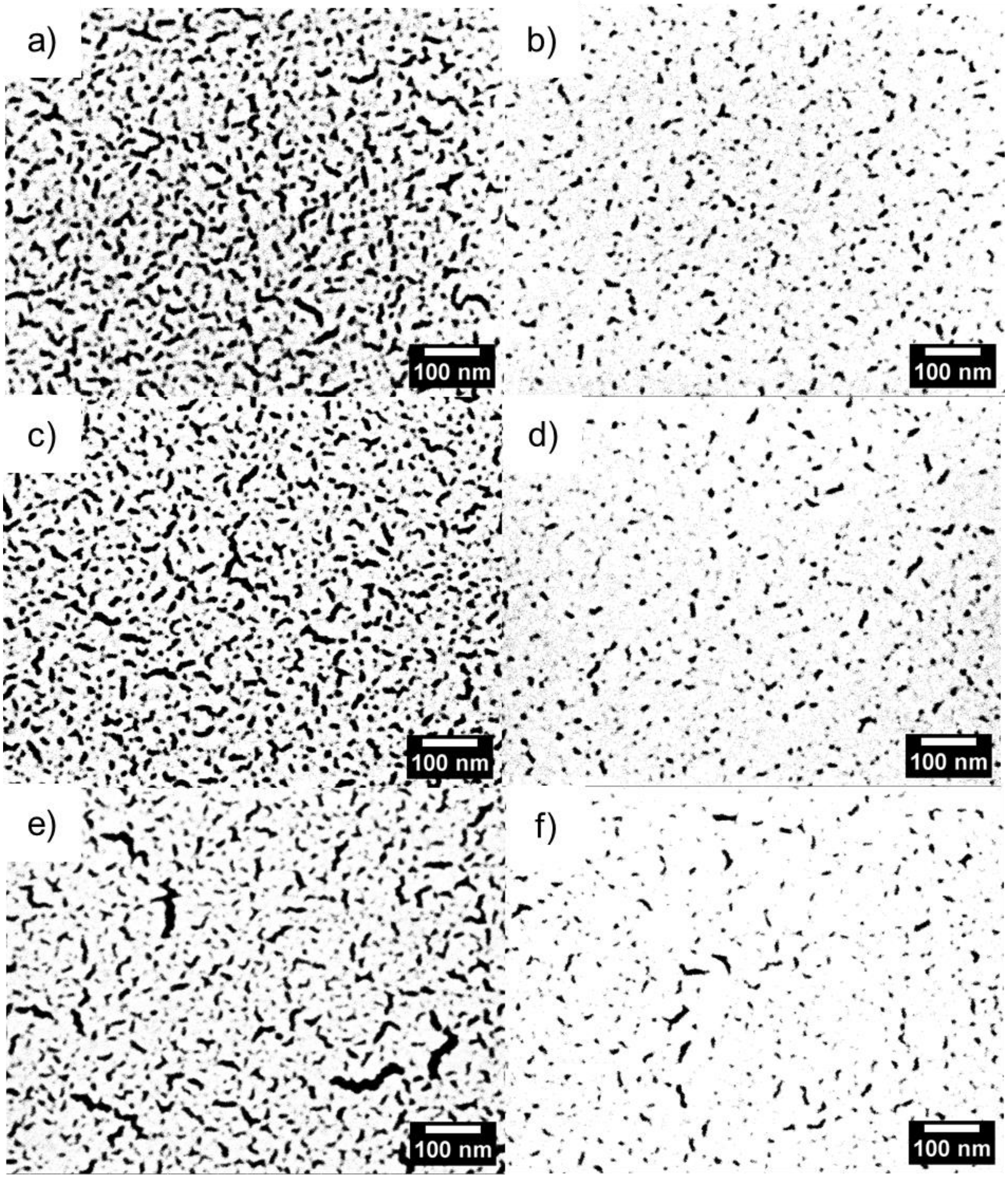

Figure S19: FESEM images of (a,c,e) dcMS and (b,d,f) HiPIMS deposited gold (Au) on (a,b) PS, (c,d) P4VP and (e,f) PSS, which were used to determine the coverage of the granular gold network at δ_{Au} = 4 nm. The FESEM images were analyzed with ImageJ and the contrast was enhanced separating regions with Au and regions without Au. A threshold was defined and utilized as described in the following. The pixel of the exposed polymer was read out and set as a new 0.

Afterwards, the contrast was enhanced to obtain FESEM images to read out the coverage. The color white stands for the percolated granular gold network and black stands for areas in which no gold coverage is apparent.

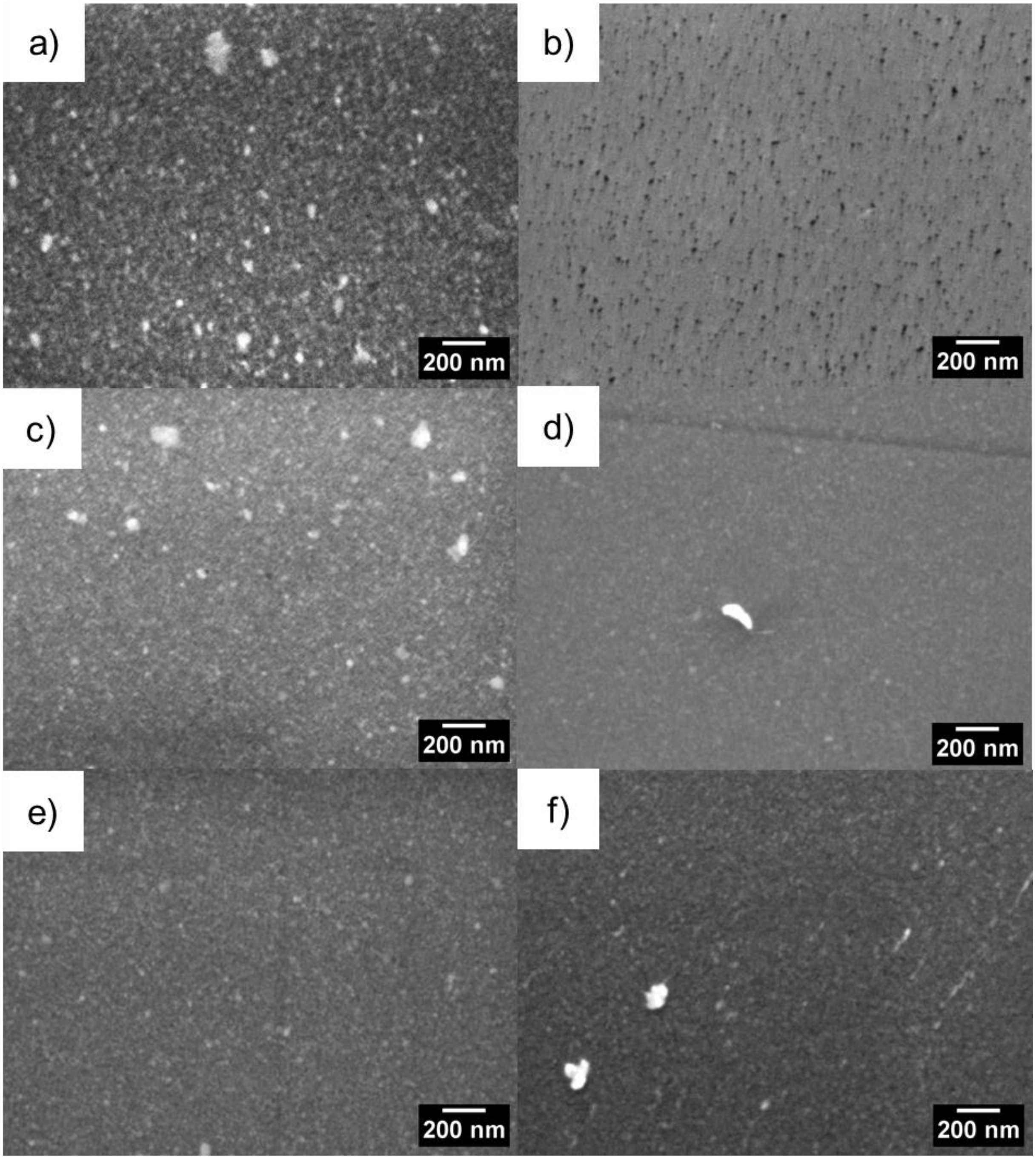

Figure S20: FESEM images of (a,c,e) dcMS and (b,d,f) HiPIMS deposited Au at a deposited thickness of δ_{Au} = 8 nm for (a,b) PS, (c,d) P4VP and (e,f) PSS.

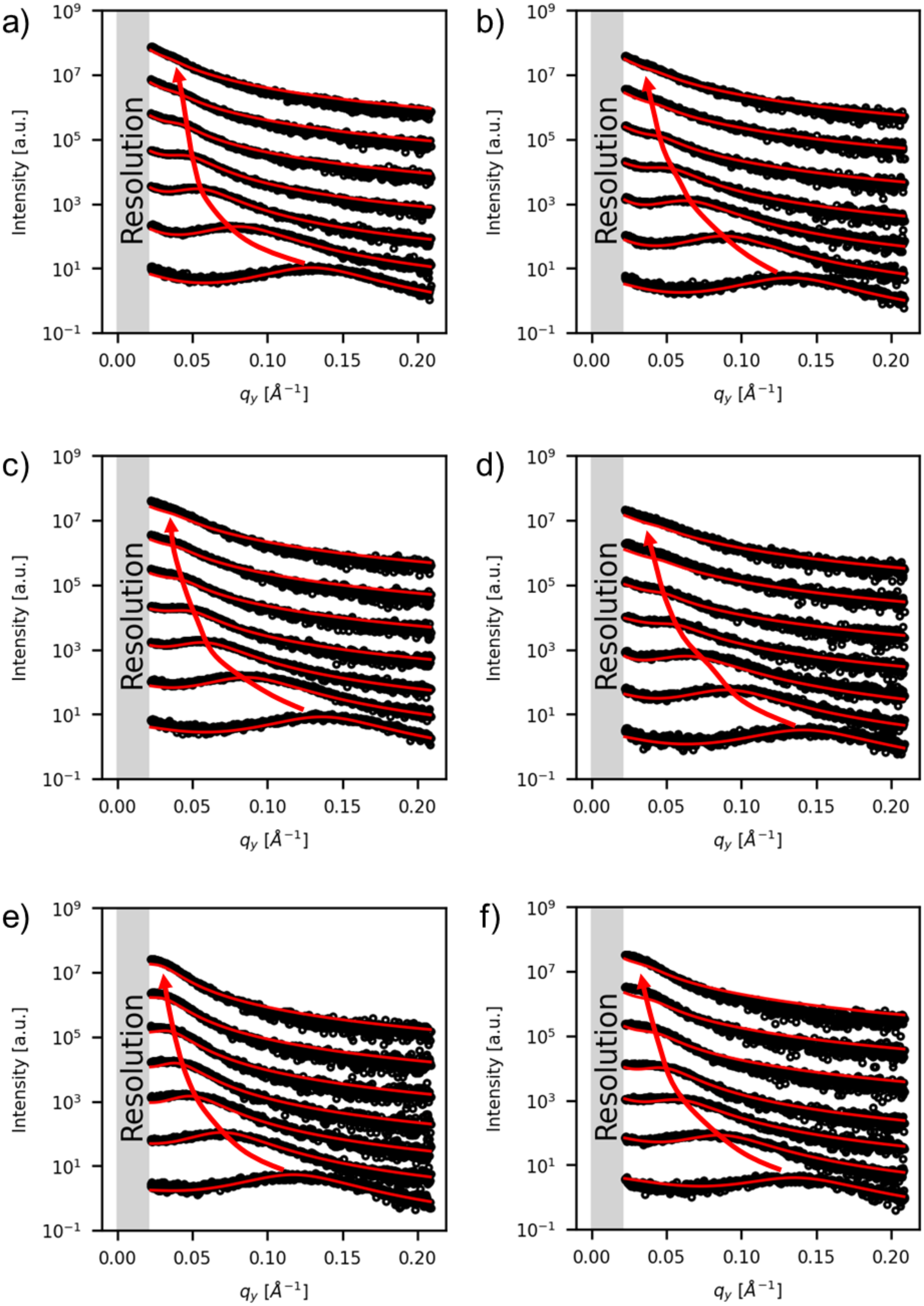
a)
b)
c)
d)
e)
f)
Resolution
Intensity [a.u.]
q_y [$Å^{-1}$]
10^9
10^7
10^5
10^3
10^1
10^{-1}
0.00
0.05
0.10
0.15
0.20

Figure S21: Horizontal line cuts from the 2D GISAXS data of the in-situ evolution of (a,c,e) dcMS and (b,d,f) HiPIMS deposited Au on (a,b) PS, (c,d) P4VP and (e,f) PSS with δ_{Au} = 1 nm, 2nm, 3nm, 4 nm, 5 nm, 6 nm and 7 nm. In every graph the bottom line cut is taken at δ_{Au} = 1 nm and is the top on at fit to δ_{Au} = 7 nm. The red arrows denote the evolution of peak position q_i. For the fit model used here for the in-situ real time GISAXS investigation the model by Schwartkopf et al. was used which consist of one Lorentzian describing the resolution function and one Lorentzian describing the peak position of the growing granular gold layer.[3]

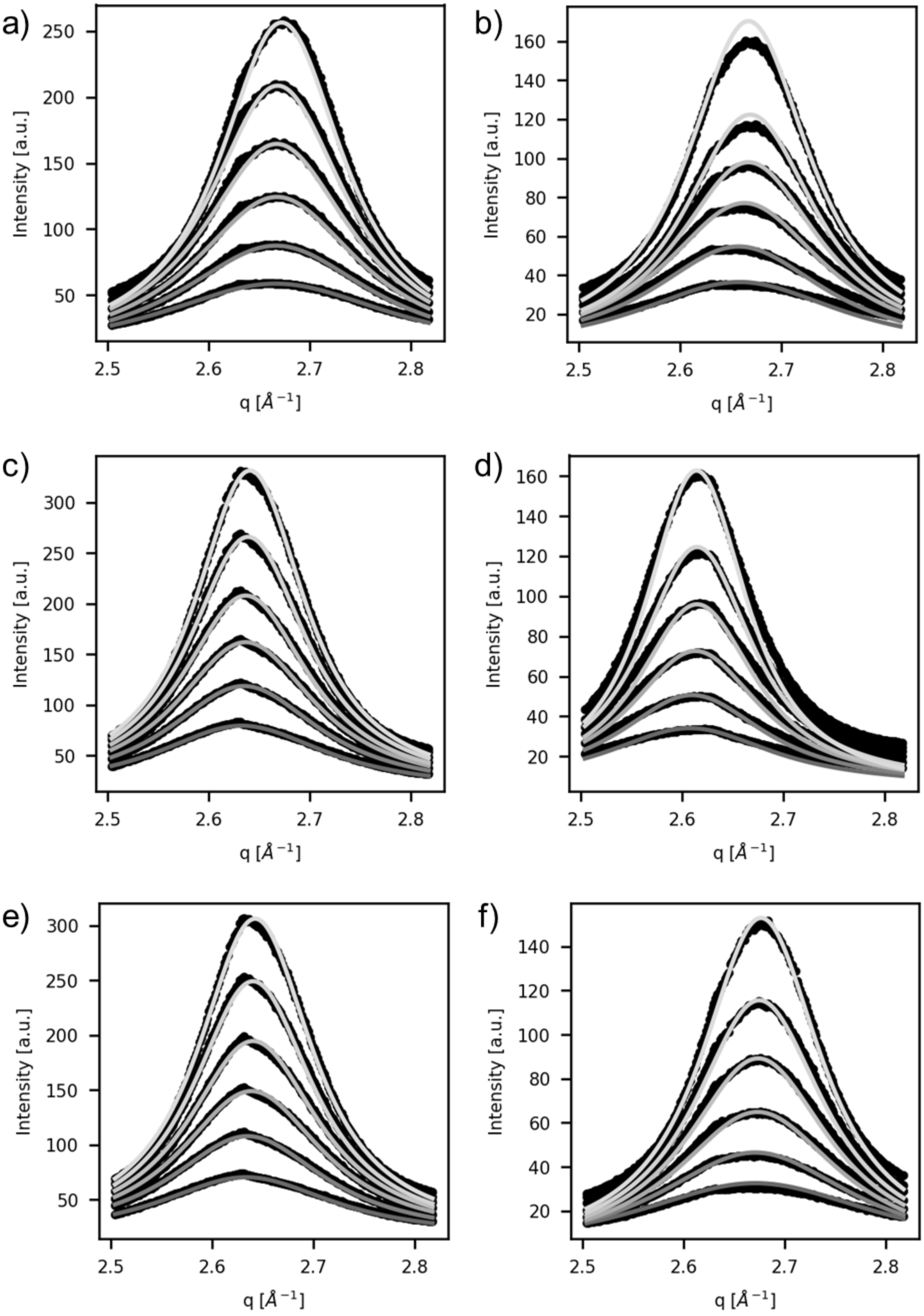
a)
b)
c)
d)
e)
f)
Intensity [a.u.]
q [Å^{-1}]

Figure S22: Pseudo-XRD extracted from 2D GIWAXS detector images of the in-situ evolution of (a,c,e) dcMS and (b,d,f) HiPIMS deposited (a,b) PS, (c,d) P4VP and (e,f) PSS of δ_{Au} = 2nm, 3nm, 4 nm, 5 nm, 6 nm and 7 nm. The integration range of the GIWAXS data was on the first diffraction ring corresponding to diffraction on Au <111> plane according to Wheeler et al.[4]

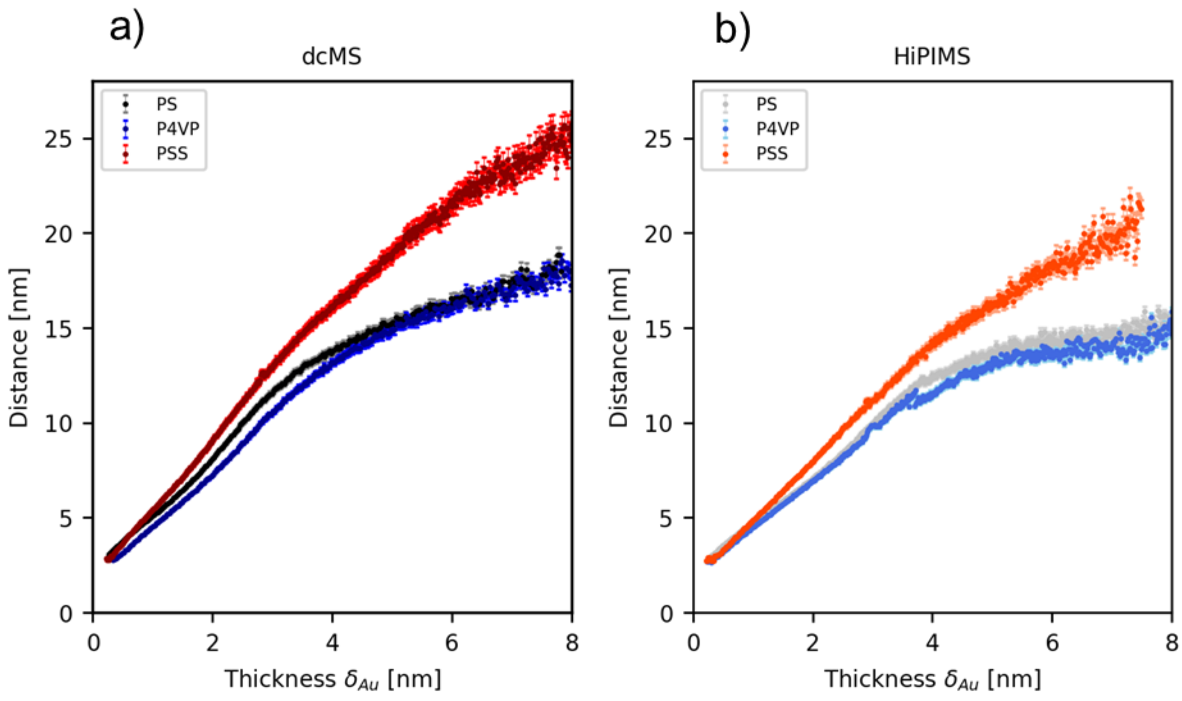

Figure S23: In-situ evolution of the distance of Au cluster deposited with (a) dcMS and (b) HiPIMS on PS (black), P4VP (blue) and PSS (red).

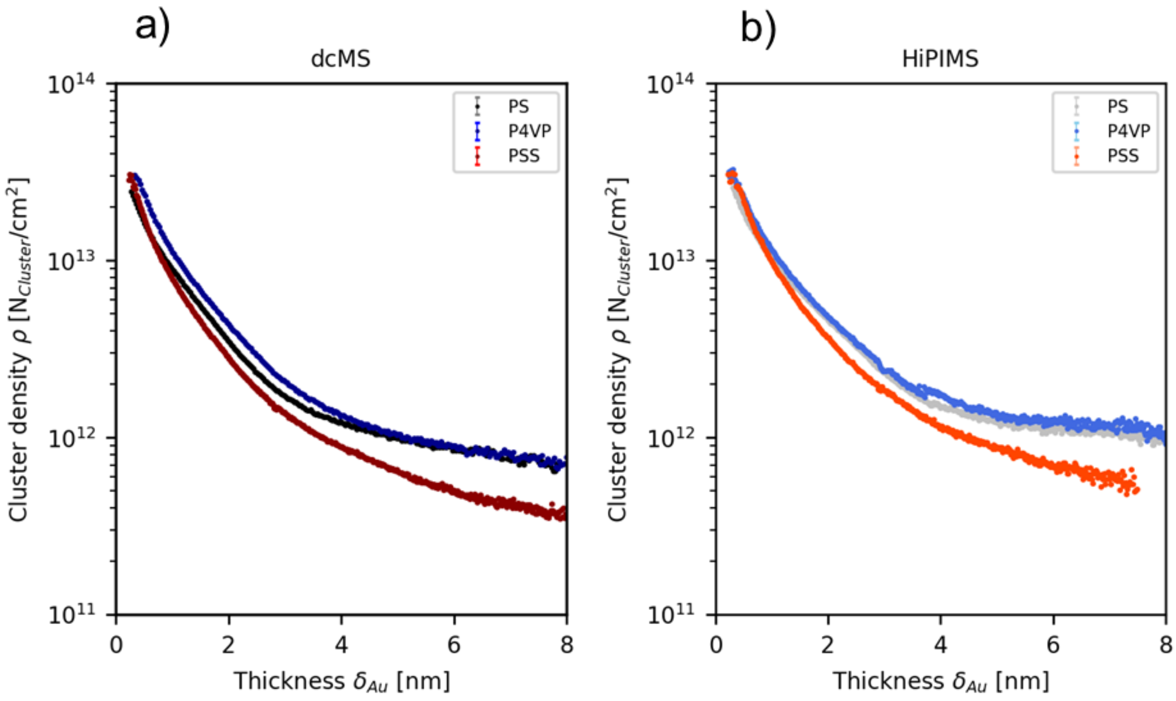

Figure S24: In situ evolution of the cluster density of Au clusters with deposited with (a) dcMS and (b) HiPIMS on PS (black), P4VP (blue) and PSS (red).

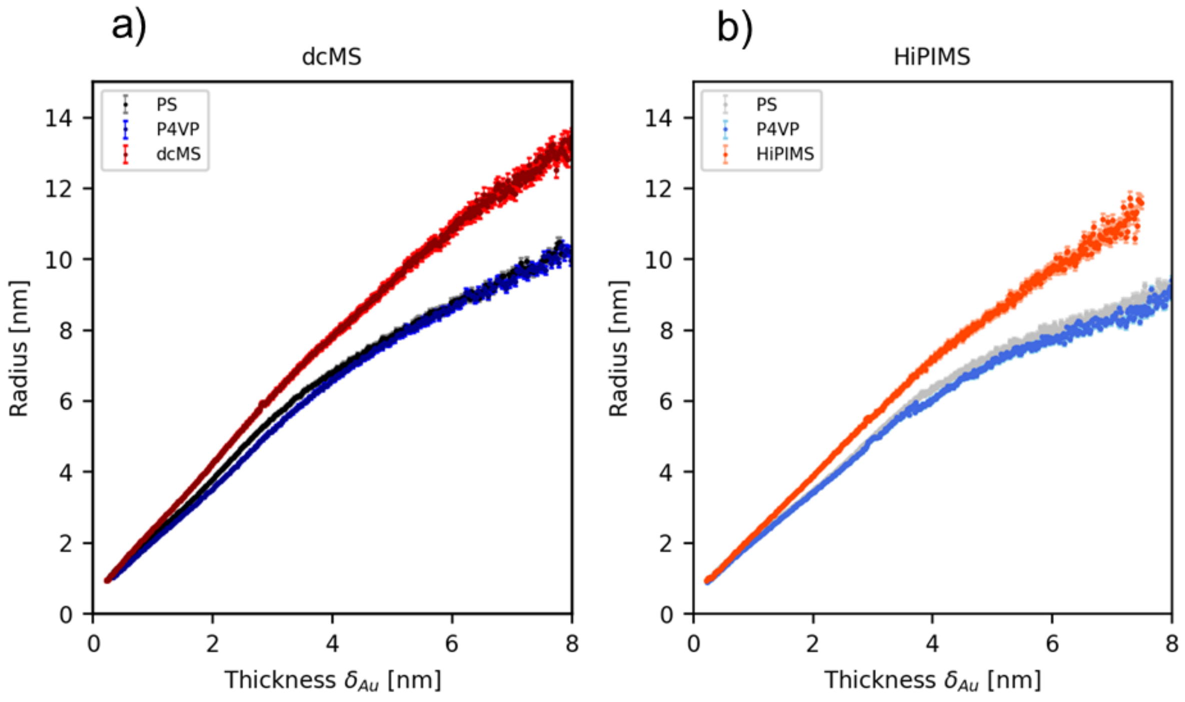

Figure S25: In-situ evolution of the radius derived from the hemispherical geometrical model deposited with (a) dcMS and (b) HiPIMS on PS (black), P4VP (blue) and PSS (red).

Table S1: Comparison of cluster density and radius from GISAXS and FESEM at δ_{Au} = 2 nm. The density of cluster was extracted by a 100 nm x 100 nm area of FESEM images in Figure 1. For the FESEM radius determination only isolated Au clusters were considered.

Material	Method	Cluster density [$N_{Clusters}$/100x100 nm^2]	Radius [nm]
PS - dcMS	GISAXS	4 ± 1	3.9 ± 0.1
PS - dcMS	FESEM	17 ± 3	2.0 ± 1.0
PS -HiPIMS	GISAXS	5 ± 1	3.5 ± 0.1
PS - HiPIMS	FESEM	23 ± 4	2.0 ± 1.0
P4VP - dcMS	GISAXS	4 ± 1	3.5 ± 0.1
P4VP - dcMS	FESEM	20 ± 3	2.0 ± 1.0
P4VP - HiPIMS	GISAXS	5 ± 1	3.4 ± 0.1
P4VP - HiPIMS	FESEM	23 ± 4	2.0 ± 1.0
PSS - dcMS	GISAXS	3 ± 1	4.1 ± 0.1
PSS - dcMS	FESEM	16 ± 3	2.0 ± 1.0
PSS- HiPIMS	GISAXS	3 ± 1	3.7 ± 0.1
PSS- HiPIMS	FESEM	19 ± 3	2.0 ± 1.0

Table S2: Summary of 4-point-probe measurement after direct fabrication and the long-term exposure being 578 days. The samples were stored under ambient conditions and in a closed cabinet. For real-world applications, a protective coating would be applied to reduce this effect.[5]

Material	Thickness (δ_{Au}) [nm]	Sheet resistance [Ω/□]			
		dcMS	578 days later	HiPIMS	578 days later
PS	3	93.8 ± 0.1	266.4 ± 0.1	53.3 ± 0.1	104.9 ± 0.1
	4	31.5 ± 0.1	64.6 ± 0.1	25.5 ± 0.1	39.6 ± 0.1
	6	16.2 ± 0.1	17.7 ± 0.1	14.0 ± 0.1	19.3 ± 0.1
	8	9.9 ± 0.1	32.3 ± 0.1	9.3 ± 0.1	30.2 ± 0.1
P4VP	3	126.5 ± 0.1	169.7 ± 0.2	55.6 ± 0.1	252.7 ± 0.1
	4	37.7 ± 0.1	67.1 ± 0.1	24.1 ± 0.1	35.6 ± 0.1
	6	16.1 ± 0.1	17.9 ± 0.1	12.8 ± 0.1	15.00 ± 0.1
	8	10.9 ± 0.1	17.0 ± 0.1	8.7 ± 0.1	11.4 ± 0.1
PSS	3	120.4 ± 0.1	367.9 ± 0.1	72.6 ± 0.1	161.4 ± 0.1
	4	49.2 ± 0.1	81.00 ± 0.1	28.1 ± 0.1	45.2 ± 0.1
	6	16.1 ± 0.1	18.1 ± 0.1	12.4 ± 0.1	15.7 ± 0.1
	8	11.5 ± 0.1	19.4 ± 0.1	8.6 ± 0.1	13.5 ± 0.1

References:

(1) Nelson, A. R. J.; Prescott, S. W. Refnx: Neutron and X-Ray Reflectometry Analysis in Python. *J. Appl. Crystallogr.* **2019**, *52*, 193–200. https://doi.org/10.1107/S1600576718017296.

(2) Amarandei, G.; Clancy, I.; O'Dwyer, C.; Arshak, A.; Corcoran, D. Stability of Ultrathin Nanocomposite Polymer Films Controlled by the Embedding of Gold Nanoparticles. *ACS Appl. Mater. Interfaces* **2014**, *6* (23), 20758–20767. https://doi.org/10.1021/am5049543.

(3) Schwartzkopf, M.; Buffet, A.; Körstgens, V.; Metwalli, E.; Schlage, K.; Benecke, G.; Perlich, J.; Rawolle, M.; Rothkirch, A.; Heidmann, B.; Herzog, G.; Müller-Buschbaum, P.; Röhlsberger, R.; Gehrke, R.; Stribeck, N.; Roth, S. V. From Atoms to Layers: In Situ Gold Cluster Growth Kinetics during Sputter Deposition. *Nanoscale* **2013**, *5* (11), 5053–5062. https://doi.org/10.1039/c3nr34216f.

(4) Davey, W. P. Precision Measurements of the Lattice Constants of Twelve Common Metals. *Phys. Rev.* **1925**, *25* (6), 753–761. https://doi.org/10.1103/PhysRev.25.753.

(5) Bauer, G.; Hassmann, J.; Walter, H.; Haglmüller, J.; Mayer, C.; Schalkhammer, T. Resonant Nanocluster Technology - From Optical Coding and High Quality Security Features to Biochips. *Nanotechnology* **2003**, *14* (12), 1289–1311. https://doi.org/10.1088/0957-4484/14/12/010.
